# *In situ* analysis of surface composition and meteorology at the Zhurong landing site on Mars

**DOI:** 10.1093/nsr/nwad056

**Published:** 2023-03-03

**Authors:** Yu-Yan Sara Zhao, Jin Yu, Guangfei Wei, Lu Pan, Xiangfeng Liu, Yangting Lin, Yang Liu, Chen Sun, Xiyu Wang, Juntao Wang, Weijie Xu, Yunfei Rao, Weiming Xu, Tianyang Sun, Fengye Chen, Beiyi Zhang, Honglei Lin, Zhenqiang Zhang, Sen Hu, Xiang-Yu Li, Xiao-Wen Yu, Shuai-Yi Qu, Di-Sheng Zhou, Xing Wu, Xiaojia Zeng, Xiongyao Li, Hong Tang, Jianzhong Liu

**Affiliations:** Research Center for Planetary Science, College of Earth Science, Chengdu University of Technology, Chengdu 610059, China; Center for Lunar and Planetary Sciences, Institute of Geochemistry, Chinese Academy of Sciences, Guiyang 550081, China; CAS Center for Excellence in Comparative Planetology, Hefei 230026, China; School of Physics and Astronomy, Shanghai Jiao Tong University, Shanghai 200240, China; Deep Space Exploration Laboratory, Hefei 230026, China; Deep Space Exploration Laboratory, Hefei 230026, China; Laboratory of Seismology and Physics of Earth's Interior, School of Earth and Space Sciences, University of Science and Technology of China, Hefei 230026, China; Key Laboratory of Space Active Opto-electronics Technology, Shanghai Institute of Technical Physics, Chinese Academy of Sciences, Shanghai 200083, China; Key Laboratory of Earth and Planetary Physics, Institute of Geology and Geophysics, Chinese Academy of Sciences, Beijing 100029, China; State Key Laboratory of Space Weather, National Space Science Center, Chinese Academy of Sciences, Beijing 100190, China; School of Physics and Astronomy, Shanghai Jiao Tong University, Shanghai 200240, China; Center for Lunar and Planetary Sciences, Institute of Geochemistry, Chinese Academy of Sciences, Guiyang 550081, China; University of Chinese Academy of Sciences, Beijing 100049, China; Center for Lunar and Planetary Sciences, Institute of Geochemistry, Chinese Academy of Sciences, Guiyang 550081, China; University of Chinese Academy of Sciences, Beijing 100049, China; School of Physics and Astronomy, Shanghai Jiao Tong University, Shanghai 200240, China; School of Physics and Astronomy, Shanghai Jiao Tong University, Shanghai 200240, China; Key Laboratory of Space Active Opto-electronics Technology, Shanghai Institute of Technical Physics, Chinese Academy of Sciences, Shanghai 200083, China; School of Physics and Astronomy, Shanghai Jiao Tong University, Shanghai 200240, China; School of Physics and Astronomy, Shanghai Jiao Tong University, Shanghai 200240, China; School of Physics and Astronomy, Shanghai Jiao Tong University, Shanghai 200240, China; Key Laboratory of Earth and Planetary Physics, Institute of Geology and Geophysics, Chinese Academy of Sciences, Beijing 100029, China; Key Laboratory of Space Active Opto-electronics Technology, Shanghai Institute of Technical Physics, Chinese Academy of Sciences, Shanghai 200083, China; Key Laboratory of Earth and Planetary Physics, Institute of Geology and Geophysics, Chinese Academy of Sciences, Beijing 100029, China; Research Center for Planetary Science, College of Earth Science, Chengdu University of Technology, Chengdu 610059, China; Center for Lunar and Planetary Sciences, Institute of Geochemistry, Chinese Academy of Sciences, Guiyang 550081, China; Center for Lunar and Planetary Sciences, Institute of Geochemistry, Chinese Academy of Sciences, Guiyang 550081, China; University of Chinese Academy of Sciences, Beijing 100049, China; Center for Lunar and Planetary Sciences, Institute of Geochemistry, Chinese Academy of Sciences, Guiyang 550081, China; University of Chinese Academy of Sciences, Beijing 100049, China; Center for Lunar and Planetary Sciences, Institute of Geochemistry, Chinese Academy of Sciences, Guiyang 550081, China; University of Chinese Academy of Sciences, Beijing 100049, China; State Key Laboratory of Space Weather, National Space Science Center, Chinese Academy of Sciences, Beijing 100190, China; Center for Lunar and Planetary Sciences, Institute of Geochemistry, Chinese Academy of Sciences, Guiyang 550081, China; CAS Center for Excellence in Comparative Planetology, Hefei 230026, China; Center for Lunar and Planetary Sciences, Institute of Geochemistry, Chinese Academy of Sciences, Guiyang 550081, China; CAS Center for Excellence in Comparative Planetology, Hefei 230026, China; Center for Lunar and Planetary Sciences, Institute of Geochemistry, Chinese Academy of Sciences, Guiyang 550081, China; CAS Center for Excellence in Comparative Planetology, Hefei 230026, China; Center for Lunar and Planetary Sciences, Institute of Geochemistry, Chinese Academy of Sciences, Guiyang 550081, China; CAS Center for Excellence in Comparative Planetology, Hefei 230026, China

**Keywords:** Zhurong rover, Tianwen-1 mission, Utopia Planitia, LIBS, volatile

## Abstract

The Zhurong rover of the Tianwen-1 mission landed in southern Utopia Planitia, providing a unique window into the evolutionary history of the Martian lowlands. During its first 110 sols, Zhurong investigated and categorized surface targets into igneous rocks, lithified duricrusts, cemented duricrusts, soils and sands. The lithified duricrusts, analysed by using laser-induced breakdown spectroscopy onboard Zhurong, show elevated water contents and distinct compositions from those of igneous rocks. The cemented duricrusts are likely formed via water vapor–frost cycling at the atmosphere–soil interface, as supported by the local meteorological conditions. Soils and sands contain elevated magnesium and water, attributed to both hydrated magnesium salts and adsorbed water. The compositional and meteorological evidence indicates potential Amazonian brine activities and present-day water vapor cycling at the soil–atmosphere interface. Searching for further clues to water-related activities and determining the water source by Zhurong are critical to constrain the volatile evolution history at the landing site.

## Teaser

The Zhurong rover finds in situ evidence for both Amazonian saline water activities and present-day water vapor cycling in the Martian northern lowlands.

## INTRODUCTION

**Figure 1.**
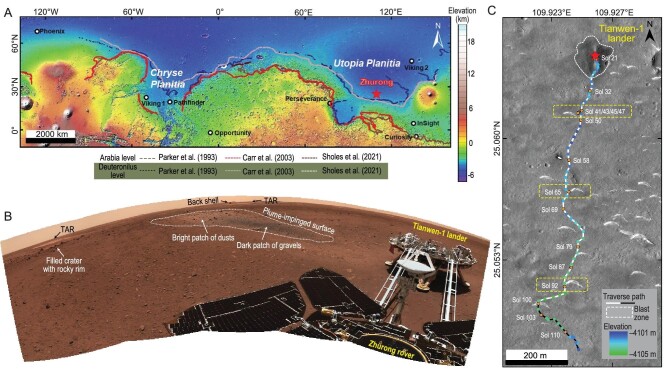
An overview of the landing site of the Zhurong rover, Tianwen-1 mission. (A) Regional context of the Zhurong landing site (marked by a star), with previous lander and rover missions within the latitude region (marked by circles). The base map uses the color hill-shade topographic data set from the Mars Orbital Laser Altimeter (MOLA). Dashed curves show the proposed and putative shorelines from the literature at the dichotomous boundary of Mars [40–42]. (B) The panorama of the touchdown site is composed of stitched images taken by the NaTeCams of the Zhurong rover. The Tianwen-1 lander and the Zhurong rover are shown to the right of the image. On the far horizon, a crater with a rocky rim, two TARs and the back shell of the Tianwen-1 lander can be identified. The descending plumes impinged the surface and removed dusts initially covering the surface, revealing a dark patch of gravels underneath the dust cover. The surface is mainly composed of gravels and clasts of rocks, soils and dust. The IDs of the NaTeCam and TMI images are tabulated in Supplementary Table S2. (C) Traverse path (white and black curves) during the first 110 sols (1130 m) since the landing on 15 May 2021. The base map is the HiRISE image (ESP_069 731_2055_RED). The red star marks the touchdown location and the white dashed lines outline the blast zone during landing. The Sol-100 marks the northern summer solstice (Ls = 90°), so the white curves from touchdown location to Sol 100 represent traversal during northern spring and the consequent traverse path in black curves represents northern summer. Blue to green dots show the elevation along the traverse extracted from the high-precision digital elevation model (DEM, DTEEC_069 665_2055_069 731_2055_A01) derived from the HiRISE image. Orange spots along the curves highlight the sols where MarSCoDe conducted the analysis.

As a part of the Tianwen-1 mission (TW-1), the Zhurong rover successfully landed at southern Utopia Planitia (109.925°E, 25.066°N; elevation of −4099.4 m) on 15 May 2021 (UTM + 8), north of the dichotomous boundary of Mars [1] (Fig. 1A). Previous orbital surveys of the landing site suggest that the underlying Vastitas Borealis Formation (VBF) was deposited during the late Hesperian (estimated ∼3.1–3.63 Ga) [2–6] and was subject to subsequent long-term modifications and resurfacing and a younger surface age of Amazonian age (∼757 Ma) was also proposed for the local area [6]. The VBF materials are considered volatile-rich, particularly water- or ice-rich, and hypothesized to be residual sedimentary deposits, indicating putative aqueous activities at the levels of glacial, fluvial, lacustrine or even oceanic activities in the Martian northern lowlands [*7*]. The relatively low-elevation and low-latitude location of the landing site of Zhurong suggest that Martian water may be stable on the surface for a longer time [8]. Therefore, the geologic history and environmental conditions potentially imply a rich aqueous-related history at this site. *In situ* observations by the Zhurong rover are expected to provide new constraints and critical insights into the role of volatiles in shaping the rocks and soils on the northern lowlands of Mars [1,9].

Here we present a comprehensive investigation on the surface compositions and meteorological conditions at the landing site. The results of Navigation and Terrain Cameras (NaTeCams) [10], laser-induced breakdown spectroscopy (LIBS) and telescopic micro-imager (TMI) of the Mars Surface Composition Detector (MarSCoDe) suite [11] and Mars Climate Station (MCS) [12] on board the Zhurong rover are integrated for indications of the geological processes and potential water vapor cycling in the local area.

## RESULTS

### Landing site environment and geological targets along the traverse

Upon touchdown, the Zhurong rover acquired panoramic images using the NaTeCams with rocks, soil, transverse aeolian ridges (TARs) and small craters in the vicinity (Fig. 1B). The engine plumes of the lander produced symmetrical bright stripes of dust in a north–south direction (Fig. 1C). Removing the bright-toned dust covering the ground surface revealed a patch of dark-toned gravels and clasts with irregular shapes (Fig. 1B). The retrorocket also excavated a hole underneath the Tianwen-1 lander and exposed an indurated layer on the ground surface [3]. The rock abundance in the landing area was estimated to be <7% from the orbit and ∼4% after the landing [3]. These dark rock clasts scattered at the landing site may be igneous rocks in nature (presumably basalts). In the subsequent roving up to 110 sols, the Zhurong rover traveled southwards for 1130 m (Fig. 1C). The rover tracks continuously reveal dark-toned materials beneath the topsoil (Supplementary Fig. S1). The rover wheel sinkage was estimated to be 5–10 mm (range 2∼15 mm) during Zhurong's first 60 sols traverse [13], suggesting that dark-toned igneous materials may blanket the local area beneath the soil cover of ≤15 mm.

**Figure 2.**
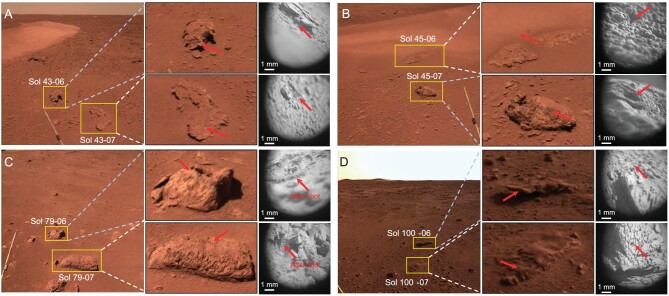
Representative rock targets observed and investigated during the 110-sol traverse. In subfigures (A–D), yellow rectangles or circles mark the targets selected in the NaTeCam images and consequently zoom in to the specific sampling spots by the LIBS marked by the red arrows and the micro-images. The sample names consist of the sol numbers and identification numbers listed in Supplementary Table S1. Note that although aiming to rock targets, most sampling spots are actually on dust/soil or cements coating the rock surface. (A) Two rock fragments analysed on Sol 43. Sample ‘Sol 43-06’ was a layered cement coating on the rock surface; sample ‘Sol 43-07’ was the dust/soils covering the rock surface. (B) Sample ‘Sol 45-06’ was the TAR sand at the foot of the eolian bedforms. Sample ‘Sol 45-07’ was measured on the rock materials indicated by an ablation pit after laser ablation. This is the only LIBS measurement of igneous rock during the first 110 sols and weathering rinds might be included in the collected spectra. (C) Two rock fragments measured on Sol 79. The LIBS laser hit the cemented coating on the rock surface and smashed the cements after ablation. (D) Two lithified duricrust samples analysed on Sol 100. Both samples show jagged etched with harder strength compared with the cements after laser ablation. The spectra of sample ‘Sol 100-06’ cannot be used for composition quantification. Micro-images were taken only after LIBS measurements before Sol 58 and, after that, micro-images both before and after LIBS measurements were available.

Rocks larger than gravels and clasts (i.e. a few centimeters to decimeters in size) were scattered along Zhurong's traverse. Dark-toned and angular float rocks similar to those of dark-toned clasts are found mostly distributed around rims of small craters (Supplementary Fig. S2A), indicating a possible origin relevant to impact processes as ejecta excavated from the subsurface. Pitted rocks, showing subangular or subrounded shapes and similar to those observed at the Viking-1 (VL1) and Viking-2 (VL2) landing sites, are primarily identified close to the touchdown site and are rarely observed in the subsequent traverse (Supplementary Fig. S2B–D). Some bright-toned rocks are also observed, either sitting on top of the regolith or partially buried (Fig. 2A–C and Supplementary Fig. S2E and F). The ventifacts and grooves on the rock surface and subrounded morphology suggest that these rocks are hard and subject to aeolian reworking. The rock surfaces that are not coated by the dust or soils reveal dark-toned interiors (Fig. 2B)and C, and Supplementary Fig. S2E and F). In line with previous *in situ* observations [14], an intriguing occurrence of rocks proposed to be lithified duricrusts was also noticed (Fig. 2D). These rocks first appeared sporadically near the touchdown site but were more widely present after Sol 34 (Supplementary Fig. S3). They were present as low-relief pavers or as thin slabs partially buried in the regolith and share similar occurrence and morphology to those imaged previously only at the Viking 1 site (Supplementary Fig. S4), although the Viking lander was unable to characterize the composition of this distinct rock at that time.

### Microscale characteristics of geological targets

The TMI and LIBS of MarSCoDe worked collaboratively to provide microscale features of the rock targets and associated compositions. Although LIBS hit the rock surface in only one of the inferred igneous rock targets (Fig. 2B, Sol 45-07), for most rock targets, the LIBS analysis was either on the soil/dust deposited onto the rock surface (Fig. 2B, Sol 45-06 and Supplementary Fig. S5) or the cement coatings on the rock surface (Fig. 2C and Supplementary Fig. S6).

As shown in the micro-images, the coarse-grained portions of the soils are well sorted and well rounded but generally lack sphericity, mostly with dust mixing in or covering the grain surface (Supplementary Figs S5 and S7). The average particle size of soil samples can be tentatively estimated. The resolution of the TMI is ∼0.03 mm per pixel (0.02∼0.04 mm depending on the target distance) so we limit our measurements to grains larger than ∼0.2 mm in size (∼7 MI pixels). Consequently, our results characterize grains beginning with fine to medium sand but do not represent the very fine sand to clay size fractions (grain size classification based on [15] hereinafter). The soil grain sizes at the landing size are in the range of 1.5∼2.5 mm (Supplementary Table S1), classified as very coarse sand (1∼ 2 mm) to very fine gravel (2∼4 mm). The mixed finer particles range 0.3∼0.5 mm and are classified as median sands. These *in situ* observations are very consistent with those estimated based on the albedo and empirical relationships between particle size and thermal inertia [16]. Thermal inertia and albedo values at the Zhurong site (thermal inertia 258 J m^−2^ s^−0.5^ K^−1^; albedo 0.23; [3]); therefore, the particle size on the ground was estimated to range from 270 μm to 2 mm. In general, well-sorted and well-rounded soil particles indicate that these soil particles have undergone transport, and the very coarse sand fractions might argue for a relatively local provenance. Dust accumulations onto the topsoil further suggest that the soil particles are stable *in situ* for an extended time. Aeolian processes show a limited capacity to effectively transport the surface materials.

The cement coatings on the rock surface show columnar or laminar microtextures, and are consistently shattered after LIBS laser shots indicating a friable nature different from the rocks (Supplementary Fig. S6). This type of cements (also referred as ‘indurated layers’) has been commonly identified at previous landing sites of northern lowlands, including the Viking-1, Viking-2, Pathfinder and InSight missions [17–20]. In comparison, the inferred lithified duricrust slabs show jagged and etched microtextures that might be due to differential weathering of already altered materials (Fig. 2D). LIBS laser shots produced small pits without destroying the bulk structure, suggesting a relatively harder strength than the cement coating on rock surfaces (Fig. 2D).

**Figure 3.**
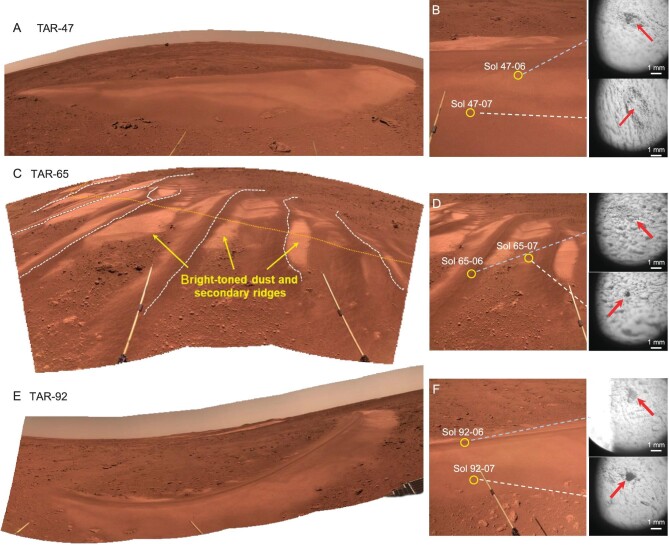
Three Eolian bedforms (TAR) observed and investigated in sequence on Sols 47, 65 and 92. (A and B) TAR-47, (C and D) TAR-65, (E and F) TAR-92. TAR-47 and TAR-92 are simple crescent-shaped deposits. TAR-65 shows prominent secondary ridges (white dashed curves) on the primary ridges (dashed curve). Bright dust has accumulated on the troughs of the aeolian bedforms and fine secondary ridges have developed on the dust deposits, indicating that TAR-65 is stationary for a long time. The IDs of the NaTeCam images are tabulated in Supplementary Table S2.

Another distinct feature in the landing area is widely distributed aeolian bedforms [13,21]. These landforms, previously identified from orbit as TARs, show high-albedo and mostly have stoss-side orienting northeastwards [13,21]. The Zhurong rover visited four TARs in sequence (i.e. T-47, T-65, T-92 and T-100; Fig. 1C) and investigated the first three with LIBS (Fig. 3). T-47 and T-92 are simple crescent deposits with a wide front and a narrow back (Fig. 1C). The bright-toned portion of the windward flank of T-47 is composed of coarse sands (grain size ∼0.6 mm; Fig. 3B and Supplementary Fig. S7L). The dark-toned portion at the TAR base is composed of very fine gravel (grain size 2.4 mm; Fig. 2B)and Supplementary Fig. S7K). The sample of TAR-92 is covered by dust and its grain size cannot be resolved (Fig. 3F)and Supplementary Fig. S7O). T-65 was different from the other two, with prominent secondary ridges developed on the deflated primary NE ridges (Fig. 3C). The dark-toned portions both on the crest of secondary ridges (2.3 mm) and in the trough (1.8 mm) are of very fine gravels and very coarse sands, respectively (Fig. 3C)and D, and Supplementary Fig. S7M and N). Bright-toned portions of TAR-65 are mainly composed of dust deposition and secondary ridges have developed on the dust surface (Fig. 3C). These observations suggest that T-65 is stationary with coarse grains armoring the surface and dust accumulation, similar to those studied *in situ* previously (e.g. Gale Crater) [22,23]. The coarse fraction grain size of bedforms at the Zhurong site is consistent with the typical grain size of inactive Rocknest and Dingo Gap bedforms (1∼3 mm) at Gale Crater [22,24,25].

### Compositions of surface targets determined by LIBS

Based on *in situ* observations, we divided the LIBS targets into five categories: igneous rocks, lithified duricrusts, cemented duricrusts, soils and TAR sands. Compositional constraints on major element (CaO, SiO_2_, Na_2_O, K_2_O, MgO, Al_2_O_3_, FeO_T_, TiO_2_) and water (H_2_O) contents in these five categories are derived from LIBS spectra using both onboard and laboratory calibrations (see Methods in Supplementary information for details). The tabulated results are listed in Supplementary Table S1. Note that the ‘igneous rocks’ category contains only one sample at which LIBS hits the rock materials rather than surface coatings (i.e. R-45-07; Fig. 2B, Sol 45-07). The ‘lithified duricrusts’ category also contains only one available sample (i.e. LD-100-07; Fig. 2G) because the analysis on another two lithified duricrust samples (i.e. LD-43-06 and LD-100-06) failed to produce usable spectra, probably because of the jagged uneven surface. The ‘cemented duricrusts’, ‘soils’ and ‘TAR sands’ categories each contain several data points and their characteristics are shown by box-whisker plots (Supplementary Fig. S8). Since LIBS measurements only represent submillimeter-scale compositions, an average of each category may better represent the general geochemical trends than individual measurements. This is particularly true for rock targets where heterogeneity is commonly present. However, due to the limited number of available data thus far, we include ‘igneous rocks’ and ‘lithified duricrusts’ in the discussion with an understanding that these results and discussion are only preliminary.

**Figure 4.**
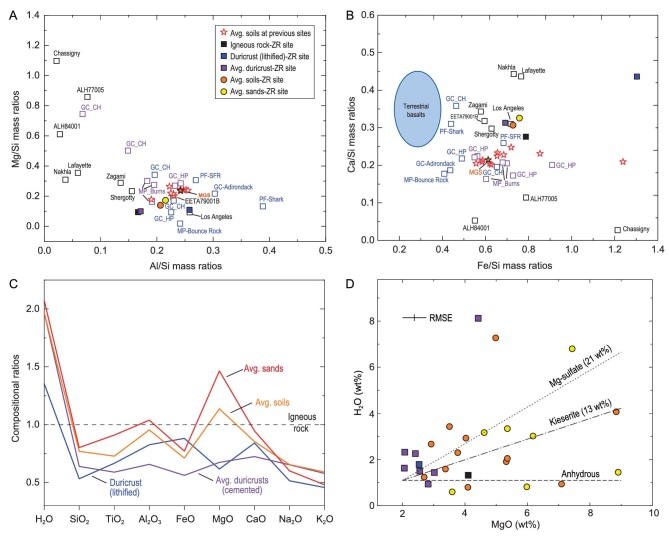
Preliminary compositions of surface materials analysed by LIBS on board the Zhurong rover. The samples are categorized into igneous rock, lithified duricrust, cemented duricrust, soils and TAR sand. (A) Mg/Si versus Al/Si mass ratios and (B) Ca/Si and Fe/Si mass ratios for terrestrial ultramafic rocks, Mars SNC meteorites, Martian meteorites and *in situ* Mars measurements (source data listed in Supplementary Table S3). The igneous rock sample at Zhurong contains less Al and higher Fe than basaltic rocks at previous landing sites. The lithified duricrust deviates from the sedimentary rocks at previous sites and contains higher Al, Ca and Fe contents. The igneous rock and lithified duricrust of the Zhurong site belong to two distinct categories. The average compositions of duricrusts, soils and sands are consistent with average soils at previous sites but with elevated Ca. In (A and B), hollow black squares represent Martian meteorites with individual names noted. The hollow blue squares represent selected basaltic rocks analysed *in situ* by using an alpha particle X-ray spectrometer. Hollow red stars each represent averaged soil compositions at previous landing sites and the solid orange star is the averaged composition of Mars Global Soil. The solid squares represent igneous rock (black), lithified duricrust (blue) and averaged cemented duricrust (purple) at the Zhurong site. The solid circles represent averaged soil (orange) and TAR sand (yellow). ‘GC’, ‘MP’ and ‘PF’ abbreviate ‘Gusev Crater’, ‘Meridiani Planum’ and ‘Pathfinder’, respectively. ‘CH’ and ‘HP’ represent ‘Columbia Hills’ and ‘Home Plate’ at Gusev Crater. ‘Burns’ represent the ‘Burns Formation’ at Meridiani Planum. ‘PF-SFR’ represents the derived soil free rock composition based on Pathfinder analysis. (C) Normalization of the other four categories of Zhurong surface materials against the igneous rock composition (Supplementary Table S4). The horizontal black line represents the igneous rock. Ratios of the average composition of lithified duricrust (blue), cemented duricrusts (purple), TAR sands (red) and soils (orange) to igneous rocks are plotted for comparison. The H_2_O contents are in the order of TAR sands > soils ≈ duricrusts > duricrust (lithified) > igneous rocks. The igneous rock has high Si, which might be due to the presence of weathered rinds on the rock surface. Soils and sands are enriched in Mg and have relatively low Na and K contents compared with local igneous rocks. The duricrusts (not lithified) generally follow the patterns of soil and sand concentrations. Errors associated with the elemental concentration data are presented in Supplementary Table S1 and are not shown here for clarity. (D) The potential correlation between the H_2_O content and MgO for different targets. Dashed and dotted lines indicate the mixing lines between a presumed endmember (MgO 2 wt% and H_2_O 1.1 wt%) and a Mg sulfate endmember with varying amounts of water indicated in parentheses. The uncertainties of the concentrations presented are estimated using the root mean square errors (RMSEs) of the calibration models (shown in Supplementary Table S1). The H_2_O-to-MgO ratios range beyond the Mg sulfates, suggesting that adsorbed water in addition to hydrated Mg sulfates may be present in these categories.

Compositional plots of Zhurong samples further confirm that the igneous rock and lithified duricrust belong to two distinct categories (Fig. 4A)and B). Compared with Martian meteorites and previous *in situ* measurements at previous landing sites, the igneous rock sample contains less Al and higher Fe than previous *in situ* basaltic rocks; the lithified duricrust sample shows higher Al, Ca and Fe, and deviates from previous *in situ* sedimentary rocks. The cemented duricrust, soil and TAR sand samples have average compositions consistent with soils at previous landing sites but relatively elevated Ca. The average compositions of soils and TAR sands presented in this study agree within error with the LIBS results reported by Liu *et al.* (2022) [26].

We normalized the other four categories against the igneous rock (i.e. R-45-07) to compare their compositional trends (Fig. 4C)and Supplementary Table S4). Geochemical trends show that igneous rock contains the highest Si abundance, which might be due to silica-rich weathering rinds that were not fully ablated by the LIBS laser. In addition, although the presumed weathering rinds are commonly considered water-rich, all other categories (including the lithified duricrusts) contain higher water abundances, in the order of TAR sands > soils ≈ cemented duricrusts > lithified duricrusts > igneous rocks. The soils and TAR sands show relative MgO enrichment and Na and K depletion compared with igneous rocks (Fig. 4C), potentially suggesting leaching and the presence of Mg salts in the soils and sands. The cemented duricrusts have similar elemental trends to sands and soils, and may share similar source materials to local soils and sands.

Further examinations of the unconsolidated categories, duricrusts, soils and sands demonstrate that H_2_O contents show no positive correlations with most major elements except for Mg (Supplementary Fig. S9). Consistently, correlations between H_2_O and H_2_O/oxide ratios suggest that H_2_O is dominant in the ratios and independent of the major cations except for partial association with the Mg phases (Supplementary Fig. S10). Assuming the elevated H_2_O and MgO are in the form of hydrated Mg sulfates as are those proposed for the Viking sites [27], however, the H_2_O-to-MgO ratios of Zhurong samples range beyond Mg sulfates (Fig. 4D). Therefore, in addition to Mg sulfates, excess water, such as adsorbed water molecules, may be present to account for the elevated H_2_O content. The presence of hydrated salts is also supported by the observed cohesive nature of Zhurong soils [13].

The high H_2_O contents in lithified duricrusts detected by LIBS are consistent with the strong OH/H_2_O signals found by the short-wavelength infrared spectroscopy of Zhurong [14]. The formation of lithified duricrust requires substantial amounts of liquid water rather than water vapor, and we prefer a scenario in which pre-depositional regolith underwent cementation and lithification during capillary evaporation with a fluctuant groundwater table [14]. This scenario is consistent with the Ground Penetrating Radar results of the Zhurong rover, suggesting that no intact lava flow or water-lain sedimentary stratigraphic column is present in the subsurface as an apparent source for the lithified duricrusts [28]. Formation depths in the subsurface and the source of water accounting for the lithified duricrust are currently unclear. We estimated a depositional thickness of <395 m for the surface materials (Supplementary Fig. S11) and the duricrusts likely formed within these depositional sediments overlying the VBF unit. However, the deflation thickness in this area cannot be constrained due to the lack of features such as rampart craters in the local area. Therefore, the lower boundary at 395 m may be the lowest depth at which lithified duricrusts would form and the formation depth could be greater if significant deflation occurred. The groundwater sources might originate from water/ice-rich materials within the depositional layer or buried VBF materials at the bottom. The type of duricrusts (e.g. gypcrete, silcrete, ferricrete) cannot be constrained due to the limited number of measurements thus far. However, unless a long-lasting hydrothermal system triggered by impacts or volcanism was present in the landing region, silcrete and ferricrete are less likely to form because their formation usually requires prolonged warm–humid conditions [29]. Nevertheless, the widely distributed lithified duricrusts for more than a kilometer traverse of Zhurong may indicate groundwater activities in a kilometer-scale area during the Amazonian.

In addition, the compositional similarity between cemented duricrusts versus soils and sands indicates a common formation process likely related to water vapor cycling on the surface at the landing site. Here, we propose that cemented duricrusts are formed by water vapor cycling at the atmosphere–soil interface through deliquescence–efflorescence- or frost–thaw-related processes. The same mechanism has been proposed for indurated layer formation at the previous Viking-2 [18] and InSight [20] landing sites. At higher pressure, transient appearances of liquid water may occur on the surface and subsequently evaporate [30], providing a mechanism for salt formation that cements the soil. Supporting data acquired by the MCS on board the Zhurong rover and numerical simulation results using the Mars Climate Database provide direct observations of the local environmental conditions and a test for this hypothesis.

### Local meteorological conditions

**Figure 5.**
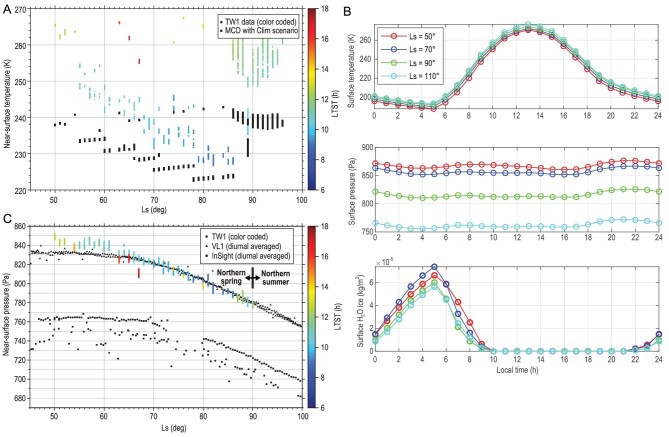
Local meteorological conditions recorded by the MCS on board Zhurong and comparison with the modeling results of the Mars Climate Database. In (A and C), the colored bars represent the measured data and solid black squares represent the simulation results at the same time and same location. The length of the colored bars represents the values of the parameters and the color indicates the measurement time at the local true solar time. (A) Near-surface temperature. The measured *T* is ∼10 K higher than the modeled results, which is well within the expected ±10 K error of the MCD simulation. (B) Simulation results of the diurnal trend of surface temperature (*T*_surf_), surface pressure and water ice contents on the ground at Ls 50° (i.e. Sol 0), 70°, 90° and 110° (i.e. Sol 137). The maximum *T*_surf_ ranges from 270.7 to 276.6 K and the minimum *T*_surf_ from 187.8 to 194.4 K. Therefore, water frost can form on the ground even during the night of the spring season and sublimate soon after sunrise. Meteorological source data from the previous Mars landing missions are available in the Data Availability Statement. (C) The air pressure data near the ground recorded by the MCS along the traverse and compared to the diurnally averaged air pressure values of Viking-1 (VL) and InSight under the same Ls conditions. TW-1, VL1 and InSight demonstrate a consistent and substantial decrease in air pressure as the northern lowlands turn from spring to summer. Note that diurnal VL1 and InSight data are averaged from day and night, and the data of the TW-1 site are only of measurements with a short duration of daytime on each sol. The data from the Pathfinder mission were not included because the mission did not contain seasonal data during Ls 50°–100°, although it was located within the latitude range and close to site VL1.

The MCS of the Zhurong rover monitored the local temperature (*T*), pressure (*P*) and wind direction and speed along the traverse [12]. The measurements were conducted during the local daytime (06:00–18:00) and lasted only for 5–50 minutes. Because measured meteorological data are in good agreement with the numerical simulations using the Mars Climate Database (MCD; v5.3) (Fig. 5 and Supplementary Figs S12 and S13), our discussion in the following is mainly based on the numerical simulation results, which enable discussion of diurnal, seasonal and annual variations in the local area given the lack of fixed local time or fixed length of the MCS *in situ* measurements.

During Ls 50°–110° (TW-1 landing and onward), the local area shows a diurnal *T*_surf_ ranging from 187.81∼193.40 to 270.70∼276.62 K, suggesting that frost of water ice would develop before the early morning and quickly disappear after sunrise (Fig. 5B). The simulated diurnal maximum *T*_surf_ (276.62 K) and air pressure (881.9 Pa) for Ls 50°–110° are higher than the triple point of water (i.e. 273.16 K, 611.73 Pa), potentially allowing a greater stability field against boiling. Because the boiling point of water is correlated with the air pressure and thus may increase with increasing surface pressure [31], examining the stability field of liquid water for pressures ranging from 600 to 900 Pa confines a narrow *T*–*P* range that can support the presence of liquid water (melting without boiling) (Supplementary Fig. S14A). Further searching for *T*–*P* conditions at the landing site that can simultaneously meet the thresholds for stability of liquid water during the entire Martian year shows that while the *P* meets the threshold for liquid water (835.1 Pa), the simulated *T* is slightly lower but close to the threshold (273.15 K) (Supplementary Fig. S14B and C). If considering the error of ±10 K in MCD simulations, transient liquid water would likely be present at the Zhurong site around the northern summer solstice. If taking the hydrated salts into account, the dissolution of salts can further depress the eutectic points to allow brines to be present.

The local meteorological conditions are primarily controlled by seasonal changes on Mars. From northern spring to summer (northern summer solstice Ls = 90° is the Sol 100 of the Zhurong traverse), *T*_surf_ and water vapor contents substantially increase (Fig. 5A)and Supplementary Fig. S12B). In contrast, the local air pressure gradually decreases (Supplementary Fig. S12A) and the dust mass in the atmosphere decreases in association with the drop in air pressure (Supplementary Fig. S12C). Simulations of the Zhurong touchdown site show annual *T* (at 1 m above the ground) ranging from 182.2 to 250.7 K, annual *P* ranging from 702.4 to 966.8 Pa and the annual water vapor column ranging from 0.010 to 0.033 kg m^−2^. In general, the Zhurong site shows a consistent seasonal trend with the Viking-1 and InSight sites (Fig. 5C). These local meteorological conditions enable water cycling and brine–salt interactions at the atmosphere–soil interfaces, which further facilitate cementation of unconsolidated materials, salt weathering on the rock surface and enrichment of hydrated salts in the soils and sands.

The local winds are primarily in the NE–SW direction, with minors in the NW direction (Supplementary Fig. S13). The primary NE–SW wind direction is consistent with the prevailing wind direction indicated by TARs [13], suggesting that NE winds may have predominated in the area since TAR formation. The measured wind speeds during the daytime are mainly 2∼6 m s^–1^, with a maximum of up to ∼10 m s^–1^ (Supplementary Fig. S13). The measured wind speeds are generally consistent with those measured at the Viking-1 and Pathfinder sites at latitudes similar to those of the northern lowlands [32].

### Comparison with previous landing sites in the northern lowlands

Before landing, the estimated thermal inertia and albedo values of the Zhurong site (258 J m^−2^ s^−0.5^ K^−1^; 0.23) are closest to landing sites such as the Viking 1 lander (292 J m^−2^ s^−0.5^ K^−1^; 0.205), Viking 2 lander (242 J m^−2^ s^−0.5^ K^−1^; 0.304) and InSight lander (218 J m^−2^ s^−0.5^ K^−1^; 0.240) [33–35], predicting surface physical properties of Zhurong similar to those previous sites in the northern lowlands except for the Pathfinder mission. Consistently, *in situ* observations confirm the similarity to the Viking 1 and 2 sites and the InSight site in general, and suggest that the Zhurong site is one of the granule- to pebble-rich landing sites on Mars [3]. The main reason for the substantial difference between the Zhurong and Pathfinder sites is that the latter was located at a depositional fan near the mouth of the catastrophic outflow channels of Ares and Tiu Vallis, and the landing site surface widely distributed meter-scale boulders, with sizes as large as 7 m [19]. Pitted rocks found at the Zhurong touchdown locations are also evident at the Viking 1 and Viking 2 sites and might argue for a similar origin involving cryogenic brine weathering processes [36]. The pulsed tetrorockets excavated the top layer of the regolith under the Tianwen-1 lander and showed the cemented and indurated layer, similar to those observed at the InSight lander site [3]. These cemented duricrusts potentially indicate brine activity and the presence of hydrated salts, which are consistent with the *in situ* observations of the Zhurong site [13,14,26] and those inferred for the Viking and InSight sites [17,18,20]. The intriguing lithified duricrusts have been inferred previously only at the Viking 1 site. The formation mechanism of lithified duricrusts and their sporadic appearance are still unclear and require further investigation. Furthermore, as shown in this study, the local meteorological conditions of a landing site are primarily controlled by the latitudes and elevations, and predominated by the seasonal variations of Mars. This indicates that different locations in the northern lowlands of Mars might have similar meteorological conditions if they share similar latitudes and elevations. At this point, due to the limited observations and measurements both at the Zhurong site and previous sites of the northern lowlands, discussion on many key phenomena and related mechanisms are largely based on inference, and further investigations are critical for further in-depth comparison and discussion.

## SUMMARY

Observations along the 110-sol traverse of the Zhurong reveal compositional and meteorological evidence indicating potentially Amazonian brine activities and present water vapor cycling at the soil–atmosphere interface. Observations of igneous clasts, eolian bedforms, lithified duricrusts and cemented duricrusts suggest diverse geological processes, including volcanism, impact and aeolian, and potentially saline water activities occurring in the Amazonian-aged units in southern Utopia Planitia. The lithified duricrusts have distinct compositions and elevated H_2_O contents compared with igneous rocks. Soils and sands contain elevated Mg and H_2_O contents, and argue for the presence of both Mg-rich hydrated salts and adsorbed water, consistently with the cohesive nature of the topsoil. The origin of Mg-salt enrichment in the local soils and sands is intriguing, and might further speculate for at least transient, Mg-enriched brines on the local surface. In addition, the cemented duricrusts, the possible existence of hydrated salts in the soils and *in situ* meteorological data point to a recently active water vapor–frost cycle at the soil–atmosphere interface at the landing site. The similar latitudes and elevations of the Zhurong and Viking 1 sites result in similar meteorological conditions on the surface and potentially account for forming the cemented duricrusts, pitted rocks and lithified duricrusts present at both sites. Continuous investigations of the Zhurong rover, particularly for additional evidence of water-related activities and clues for water sources in the landing area, are critical to constrain the volatile evolution history in the northern plains of Mars.

## METHODS

### MarSCoDe LIBS data processing and quantitative analysis

The Mars Surface Composition Detector (MarSCoDe) is a major scientific payload equipped on the Zhurong rover [11]. This remote sensing instrument suite uses LIBS for active spectroscopic analyses over a spectral range of 240–850 nm, shortwave infrared spectroscopy (SWIR) for passive spectroscopic analyses over a spectral range of 800–2400 nm and a TMI to capture texture and morphology images of the samples, all with a stand-off distance from 1.6 to 7.0 m [11].

LIBS spectra processed in this work were acquired during the first 110 sols, including a total of 45 calibration (CAL) spectra and 32 scientific (SCI) spectra. A list of *in situ* LIBS spectra processed in this study is presented in Supplementary Table S5. LIBS spectra of the 32 SCI targets, five *in situ* Norite calibration target measurements and four igneous reference samples used for quantifying H are shown in Supplementary Fig. S15. Note that only the five spectra of the Norite onboard calibration target [11] were used to establish calibration models of the eight major oxides determined by LIBS. The preprocessed data in Level 2 were produced and delivered by the Key Laboratory of Lunar and Deep Space Exploration, National Astronomical Observatories, Chinese Academy of Sciences [37]. The flowchart of the LIBS data treatment processes used in this study is shown in Supplementary Fig. S16. The detailed methods of LIBS spectral data processing and qualitative analysis are presented in the Supplementary information.

### MCS ***in situ*** measurements

The MCS on board Tianwen-1 is designed to measure local near-surface atmospheric temperature, pressure, wind and sound on the Martian surface [12]. The MCS consists of four units: Measurements Unit 1 (MU1) and Unit 2 (MU2), which are installed outside of the Zhurong rover to be exposed to the atmosphere; an instrument control unit (ICU), which is shielded in the rover cabin; and a cable kit, which has a function to connect the ICU with MU1 and MU2. Both the temperature and pressure sensors were installed on the rover with a height of ∼0.6 m, and the wind and microphone units were installed on the mast with a height of ∼1.5 m. The sampling frequency of MCS is 1 Hz. The details of MCS measurements are presented in the Supplementary information.

### MCD and numerical simulations

The MCD v5.3 is a database of atmospheric parameters derived from running the LMD Mars Global Climate Model (LMD GCM) [38]. The MCD can provide a high-resolution mode (32 pix/deg) based on Mars Orbital Laser Altimeter (MOLA) topography data, mean Viking Lander 1 seasonal pressure measurements and LMD GCM-derived horizontal pressure gradients. The details of the MCD simulations are presented in the Supplementary information.

### Estimation of the thickness of the surface deposits at the landing site

Ghost craters record an episode of burial that can be used to estimate the depth of the mantling deposit. We adopt the ratio of the fresh crater diameter and rim thickness studied in the Utopia Planitia [39]. The details of deposition thickness estimation are presented in the Supplementary information.

## DATA AVAILABILITY

All data needed to evaluate the conclusions in the paper are presented in the paper and the Supplementary materials. The data reported in this work are available at the Lunar and Planetary Data Release System (https://moon.bao.ac.cn/web/manager/home). Path to access the data: Home Page > Scientific Data > Mars. Meteorological data of the previous Mars landers are available at https://pds-atmospheres.nmsu.edu/data_and_services/atmospheres_data/MARS/mars_lander.html.

## Supplementary Material

nwad056_Supplemental_File
