# Supplementary material for: In situ analysis of surface composition and meteorology at the Zhurong landing site on Mars
Source: Natl Sci Rev. 2023 Mar 3;10(6):nwad056. doi: 10.1093/nsr/nwad056 (PMC10171622; doi:10.1093/nsr/nwad056)
Supplement: nwad056_Supplemental_File [file nwad056_supplemental_file.pdf]

SUPPLEMENTARY INFORMATION

In situ analysis of surface composition and meteorology at the Zhurong landing site on Mars

Yu-Yan Sara Zhao (赵宇鴳) [1,2,3]*, Jin Yu (俞进) [4]*, Guangfei Wei (魏广飞) [5,3], Lu Pan (潘路) [6,5], Xiangfeng Liu (刘向锋) [7], Yangting Lin (林杨挺) [8], Yang Liu (刘洋) [9,3], Chen Sun (孙琛) [4], Xiyu Wang (王夕予) [2,10], Juntao Wang (王俊涛) [2,10], Weijie Xu (徐伟杰) [4], Yunfei Rao (饶云飞) [4], Weiming Xu (徐卫明) [7]*, Tianyang Sun (孙天洋) [4], Fengye Chen (陈枫叶) [4], Beiyi Zhang (张倍艺) [4], Honglei Lin (林红磊) [8], Zhenqiang Zhang (张振强) [7], Sen Hu (胡森) [8], Xiang-Yu Li (李翔宇) [1,2], Xiao-Wen Yu (余晓文) [2,10], Shuai-Yi Qu (曲帅屹) [2,10], Di-Sheng Zhou (周迪圣) [2,10], Xing Wu (吴兴) [9], Xiaojia Zeng (曾小家) [2,3], Xiongyao Li (李雄耀) [2,3], Hong Tang (唐红) [2,3], Jianzhong Liu (刘建忠) [2,3]*

Affiliations:

[1]International Center for Planetary Science, College of Earth Science, Chengdu University of Technology; Chengdu 610059, China.

[2]Center for Lunar and Planetary Sciences, Institute of Geochemistry, Chinese Academy of Sciences; Guiyang 550081, China.

[3]CAS Center for Excellence in Comparative Planetology; Hefei 230026, China.

[4]School of Physics and Astronomy, Shanghai Jiao Tong University; Shanghai 200240, China.

[5]Deep Space Exploration Laboratory; Hefei 230026, China

[6]Laboratory of Seismology and Physics of Earth's Interior, School of Earth and Space Sciences, University of Science and Technology of China, Hefei 230026, China

[7]Key Laboratory of Space Active Opto-electronics Technology, Shanghai Institute of Technical Physics, Chinese Academy of Sciences; Shanghai 200083, China.

[8]Key Laboratory of Earth and Planetary Physics, Institute of Geology and Geophysics, Chinese Academy of Sciences; Beijing 100029, China.

[9]State Key Laboratory of Space Weather, National Space Science Center, Chinese Academy of Sciences; Beijing 100190, China.

[10]University of Chinese Academy of Sciences; Beijing 100049, China.

*Corresponding to Yuyan Zhao, zhaoyuyan@cdut.edu.cn; Jin Yu, jin.yu@sjtu.edu.cn; Weiming Xu, xuwm@mail.sitp.ac.cn; Jianzhong Liu, liujianzhong@vip.gyig.ac.cn

This file includes:

Methods

Supplementary Figures 1 to 19

Supplementary Tables 1 to 9

References

Methods

MarSCoDe LIBS data processing and quantitative analysis

LIBS measurements and preprocessed spectra

Mars Surface Composition Detector (MarSCoDe) is a major scientific payload equipped on the Zhurong rover in the Tianwen-1 mission, first China's Mars exploration launched in 2020 [1]. This remote sensing instrument suite uses laser-induced breakdown spectroscopy (LIBS) for active spectroscopic analyses over a spectral range of 240 - 850 nm, shortwave infrared spectroscopy (SWIR) for passive spectroscopic analyses over a spectral range of 800 - 2400 nm, and a telescopic micro-imager (TMI) to capture texture and morphology images of the samples, and thereby performs in particular, in situ measurements of the elemental composition of surface soils, rocks, and minerals on Mars, with a stand-off distance from 1.6 to 7.0 m [1].

The selection of scientifical targets was ensured by the Navigation and Terrain Camera (NaTeCam) [2]. When the Zhurong rover arrives at an interesting site for scientifical investigations, the NaTeCam takes an image, which fixes a reference frame of the space that the camera looks at. In addition, the binocular stereo cameras of the system provide a 3D determination of the coordinates in the space [2]. According to the image of the NaTeCam, a scientifical target is chosen. Its coordinates are then transmitted to the MarSCoDe instrument in order to direct the TMI and the LIBS laser (the both instruments are coaxial) to the scientifical target using the biaxial pointing mirror [1]. When a LIBS spectrum is recorded, the information about the instrument (especially its geometrical posture) and the time of the recording are saved. This information will allow corresponding each LIBS spectrum to an image of TMI, and to an image of NaTeCam, providing therefore the surface morphological information of the target and its geological context for the recorded LIBS spectrum.

LIBS spectra processed in this work were acquired during the first 110 sols, including a total of 45 calibration (CAL) spectra and 32 scientific (SCI) spectra. A list of in situ LIBS spectra processed in this study is presented in **Supplementary Table 5**. Note that only the five spectra of the Norite onboard calibration target [1] were used to establish calibration models of the eight major oxides determined by LIBS. The preprocessed data in Level 2 were produced and delivered by the Key Laboratory of Lunar and Deep Space Exploration, National Astronomical Observatories, Chinese Academy of Sciences [3], with dark count and relative radiometric corrections [1]. Each spectrum data file was associated with a text file containing in particular, three sets of four parameters, $a, b, c, \Delta d$, defining an onboard calibration function in the form of a quadratic polynomial fitting function,

$$\lambda = a(x + \Delta d)^2 + b(x + \Delta d) + c \qquad \text{(Eq. S1)}$$

for each of the three spectrometers used in MarSCoDe [1]. The onboard calibration function thus converts the spectral channels (x) of a spectrum data into wavelengths. Such onboard calibration takes into account the drift of the spectrometer due to in particular, temperature changes on Mars [4]. A titanium alloy plate is included in the Calibration Target Assembly (CTA) onboard Zhurong Rover. Its spectrum was recorded before the launch of the mission in a Mars environment simulation chamber and on Mars in situ before a measurement of scientific targets, with the MarSCoDe instrument. A comparison between the above two spectra allowed determining the sets of the parameters (especially Δd) for a precise calibration of in situ spectra. The associated text file contains also information about the distance from the rover to the target, as well as the posture of the instrument and the time of the experiment, which allowed the recorded spectrum to be correlated to the data from other instruments onboard the rover, as we mentioned above for the TMI and the NaTeCam.

LIBS spectrum processing method in this work and qualitative analysis

The preprocessed data of each LIBS spectrum included three segments of data respectively recorded by the three spectrometers covering different nominal wavelength ranges of UV (240 – 340 nm), VIS (340 – 540 nm) and NIR (540 – 850 nm) [1]. Using the onboard calibration function with the corresponding parameters, the data were associated to wavelengths, generating three spectra respectively in the above-mentioned spectral ranges. These spectra were first respectively corrected for their baseline using an algorithm of morphology-based cubic p-spline fitting [5]. The baseline-corrected spectra were then merged into an entire spectrum of the preprocessed data. In merging the three spectral segments, the spectral channels in a segment simultaneously covered by the adjacent one with longer wavelengths were removed. **Supplementary Figure 15** shows the merged baseline-corrected spectra of the 32 scientific targets and those of the Norite onboard calibration target. The spectra of the Norite target were used in this work for the determination of major oxides in scientifical targets. For water concentration determination, we used four laboratory-prepared igneous rock samples as standards, and recorded their spectra in a Mars environment simulation chamber using the MarSCoDe replicate [6]. The same protocol was applied to preprocess these spectra (**Supplementary Figure 16**). One of the calibration spectra (C1) is not valid, since presenting a constant level of zero within the observed spectral range. A check in the scientifical spectra, reveals also two invalid data (S7 and S27). Invalid spectra were excluded from further processing in this work. An example of the merged baseline-corrected spectrum (S4: HX1-Ro_GRAS_MarSCoDe-LIBS-I-06_SCI_N_20210615174910_20210615174931_00032_A.2B) of a scientific target is shown in detail in **Supplementary Figure 17**, together with in the insets, enlarged presentations of C I 247.9 nm, H I 656.3 nm and O I 777 nm lines. The lines of eight major elements used in this work for the quantification of the related oxides are also indicated in the figure.

Determination of major oxides of the scientifical targets using calibration models built with the Norite calibration spectra.

For the determination of major oxides (Na_2O, MgO, Al_2O_3, SiO_2, K_2O, CaO, TiO_2, and FeO_T) of the scientifical targets, the four valid CAL spectra of the Norite onboard calibration target was used as the standard. Its major oxide concentrations are given in **Supplementary Table 6**. To reduce the influences of a wide range of effects on the SCI and CAL spectra, they were normalized before being used for calibration models and concentration determination. The influencing effects can include the chemical and physical matrix effects due to various bulk chemistries and surface physical states of the CAL and SCI targets, as well as the variations in excitation and collection conditions occurring in the measurements, in particular, changes of detection distance and incidence angle of the laser pulse on the target. The coupled effect of these perturbations leads to a large variation of SCI as well as CAL spectra, as can be seen in **Supplementary Figures 15A and 15B**. Several normalization methods were tested, including normalizations with the detection distance and with the line intensities of the elements of ambient gas, oxygen (O I 777.2 nm line) or carbon (C I 247.9 nm line), considered as internal standards. The total spectral intensity was used as a metrics to assess the performance of the normalization processes. **Supplementary Table 7** shows statistics on the total spectral intensities of the merged baseline-corrected spectra before and after normalization, for the CAL and SCI targets.

Supplementary Table 7 shows a large dispersion for the CAL as well as the SCI spectra. In addition, a large bias can be observed for the SCI spectra with respect to the CAL spectra. With normalization, the respective dispersions of the two groups of spectra decrease, accompanied by a decrease of the bias between them. The data show an optimized performance for the normalization with the C I 247.9 nm line. The reduced relative standard deviation (RSD) of the total spectral intensities of the SCI spectra could be considered to be mainly due to the various chemical compositions of the SCI targets. The relative bias of the SCI spectra with

respect to the CAL spectra is reduced to 7.3%. **Supplementary Figure 18** shows the SCI and CAL spectra normalized by C I 247.9 nm line. A significant reduction of the dispersion among the spectra of the same group and the discrepancy between the two groups of spectra can be found, conforming the results in **Supplementary Table 7**. The spectra normalized with C I 247.9 nm line were thus used for further processing.

The four normalized CAL spectra of the Norite target were used to establish calibration models for the eight major oxides with the reference concentrations in **Supplementary Table 6.** For each element, a calibration model is built between the intensity of the representative line of the element and its concentration, by considering a straight line passing through the origin (0, 0 point of the couple of variables intensity-concentration) and the point determined by the mean value of the line intensities of the element over the four normalized CAL spectra of the Norite target and the concentration of the element. For the eight major oxides, the representative emission lines are respectively Na I 589.0 nm, Mg II 279.6 nm, Al I 394.4 nm, Si I 288.2 nm, K I 769.9 nm, Ca II 393.4 nm, Ti II 334.9 nm, and Fe II 259.9 nm. The calibration performance of the models is assessed with root mean square error of calibration (RMSEC) and mean relative error of calibration (REC) defined in the following way.

$$RMSEC = \sqrt{\frac{1}{N_{CAL}} \sum_{i=1}^{N_{CAL}} \left(\hat{C}_{CAL}^{i} - C_{CAL}^{i}\right)^2} \ , \qquad \text{(Eq. S2)}$$

$$REC(\%) = \frac{100}{N_{CAL}} \sum_{i=1}^{N_{CAL}} \frac{\left|\hat{C}_{CAL}^{i} - C_{CAL}^{i}\right|}{C_{CAL}^{i}} \ , \qquad \text{(Eq. S3)}$$

where N_{CAL} is the number of CAL spectra used, C_{CAL}^{i} stands for the reference concentration of the sample giving rise to the ith CAL spectrum, and $\hat{C}_{CAL}^{i}$ refers to the model-predicted concentration with the same spectrum. The calibration models were then used to predict the concentrations of the major oxides of the 30 SCI spectra.

Determination of water of the scientific targets using calibration models built with spectra acquired in a Mars environment simulation chamber

To determine the water content of the scientific targets, four types of igneous rocks were collected, prepared in pressed powder pellets, and characterized at the Zhe Jiang Institute of Geology and Mineral Resources (http://www.zjigm.com/index.php/Home/English/). Their water content and concentrations of major oxides are provided in **Supplementary Table 8**. Spectra were recorded using the laboratory replicate model of MarSCoDe in a Martian chamber at Hangzhou Institute for Advanced Study, UCAS (http://hias.ucas.ac.cn). The replicate model of MarSCoDe and the Martian chamber are described in detail in [6]. The replicate instrument and the prepared samples were placed in a vacuum chamber filled with gases simulating the composition of the Martian atmosphere, with 1.6% Ar, 2.7% N_2 and 95.7% CO_2 at a pressure of 814 ± 15 Pa and a temperature of -16 ± 5°C. The laser was fired at a fixed distance of 2.0 m and at a repetition rate of 3 Hz. The spectra were acquired with the same protocol as Mars in situ measurements with the same unit of three spectrometers covering the UV, VIS and NIR spectral ranges. But for this work, only the data from the VIS and NIR spectrometers were analyzed. The spectra of the scientifical targets were thus truncated to fit the spectral range of the calibration spectra.

An identical data pretreatment procedure was applied to the acquired LIBS spectra. The pretreatment spectra were further normalized and used as calibration data for a regression model between the intensity of H I 656.3 nm line and the content of water. In the absence of the C I 247.9 nm line for these spectra, the ensemble of calibration and SCI spectra were normalized with total spectral intensity (**Supplementary Figure 19**). The line intensity of O I 777.2 nm line was used as a metrics for the assessment of the normalization. The results presented in **Supplementary Table 9** show the improvement with the normalization with total spectral intensity. The four normalized calibration spectra were used to establish the calibration model for H_2O with the reference concentrations in **Supplementary Table 8**, using the

intensity of H I 656.3 nm line. The calibration curve is the least-square linear fit of the intensities of H I 656.3 nm line of the 4 normalized calibration spectra as functions of their respective water concentration. The model performance assessment parameters were calculated with RMSEC and REC. The calibration model was then used to predict the concentration of H_2O in the 30 SCI spectra.

Mars Climate Station (MCS) and in situ measurements

The Mars Climate Station (MCS) onboard China's first Mars mission, Tianwen-1 (TW-1), is designed to measure local near-surface atmospheric temperature, pressure, wind, and sound on the Martian surface [7]. The MCS consists of four units, Measurements Unit 1 (MU1) and Unit 2 (MU2), which are installed outside of the Zhurong rover to be exposed to the atmosphere; an Instrument Control Unit (ICU), which is shielded in the rover cabin; and a Cable kit, which has a function to connect ICU with MU1 and MU2. More details about the MCS instrument can be found in [7]. Both the temperature and pressure sensors were installed on the rover with a height of ~0.6 m, and the wind and microphone units were installed on the mast with a height of ~1.5 m. The sampling frequency of MCS is 1 Hz.

After the ground calibration, the measurement of temperature was in the range of 153.15-223.15 K with a resolution of 0.1 K and an accuracy of 1 K, the measurement of pressure was in the range of 1-1500 Pa with 0.1 Pa resolution and 1% accuracy, the measurement of wind speed is in the range of 0-70 m/s with the best resolution of 0.2 m/s and an accuracy of 1 m/s, and the wind direction measurement range is 0-360° with a resolution of 5° and <15° accuracy.

Mars Climate Database (v5.3) and numerical simulations

The Mars Climate Database (MCD) v5.3 is a database of atmospheric parameters derived from running the LMD GCM. The MCD can provide a high-resolution mode (32 pix/deg) based on MOLA topography data, mean Viking lander 1 seasonal pressure measurements and

LMD GCM-derived horizontal pressure gradients. Given a time and location, the MCD software can yield pressure, temperature, wind and dust deposition, for example, by linear interpolation from stored data. The MCD provides a number of different synthetic cases based on predefined dust forcing scenarios: the standard “climatology”, “warm”, “cold” and “dust storm” scenarios [8]. It is worth noting that the “climatology” dust scenario is built upon the average values observed over the years without global dust storms. In this study, we use the typical Mars year dust scenario and average solar EUV conditions and no perturbation as inputs of the MCD database. We can retrieve the water vapor column and dust mass mixing ratio at the TW-1 landing area corresponding to the same local time (LTST) of the MCS observations. Additionally, we calculate these values by inputting the Ls and LTST the same as the MCS observations as well as the longitude and latitude of the rover.

The water boiling point curve between 600 – 900 Pa was calculated along with the water melting line to evaluate if transient liquid water could present at the Zhurong site via water vapor cycling at the atmosphere-soil interface. The Therefore, we have calculated the boiling curve in the pressure range of 600 to 900 Pa based on the Clausius-Clapeyron equation (Eq. S4)

$$\ln\left(\frac{P_1}{P_2}\right) = \frac{-\Delta H_{vap}}{R}\left(\frac{1}{T_1} - \frac{1}{T_2}\right) \quad \text{(Eq. S4)}$$

where P_1 and P_2 are the vapor pressures corresponding to the temperatures T_1 and T_2, respectively, ΔH_vap is the enthalpy of vaporization, and R is the gas constant (8.3145 J mol-1 K-1). The resultant interaction of water melting line and water boiling curve confine the “liquid water zone” of which the water can remain as liquid (i.e., melting but not boiling). The resultant *T*-*P* conditions of the liquid water zone were then compared with the calculated air *T* and *P* at the Zhurong site to evaluate if the surface conditions would meet the conditions to allow for liquid water to occur.

Estimation of the thickness of the surface deposits at the landing site

Ghost crater records an episode of burial that can be used to estimate the depth of the mantling deposit. We adopt the ratio of the fresh crater diameter and rim thickness studied in the Utopia Planitia, which can eliminate the effects of target materials on the geometry of the crater. The equations

$$H_R = 0.076 D_R^{0.70} \quad D < 10\ \text{km} \qquad \text{(Eq. S5)}$$

$$H_R = 0.175 D_R^{0.33} \quad D > 10\ \text{km} \qquad \text{(Eq. S6)}$$

are used to calculate the expected rim height (H_R) for a crater given diameter (D_R). The H_R of one unnamed ghost crater and the Weijiashi ghost craters were determined to be 304 m and 447 m, respectively, using the equations S5 and S6. The MOLA topography grid data were used to measure the current rim height (HRC), which is the differential elevation between the average surrounding plains and the highest of the preserved crater rim. The HRC of the unnamed and Weijiashi ghost craters were determined to be 2 m and 52 m, respectively, using spatial zone analysis in ArcGIS Tools. The surrounding plains, or the burial surface, are always at a distance larger than one crater diameter from the rim. The calculated mantling depth (Dm) obtained by subtracting the HRC from the H_R are 302 m and 395 m, respectively, for unnamed and Weijiashi craters. Therefore, the estimated upper limit of the thickness of the overlying basal materials to be 395 m above the VBF unit. The estimated mantling thickness provides a constraint on the infilling and deposition processes in the region.

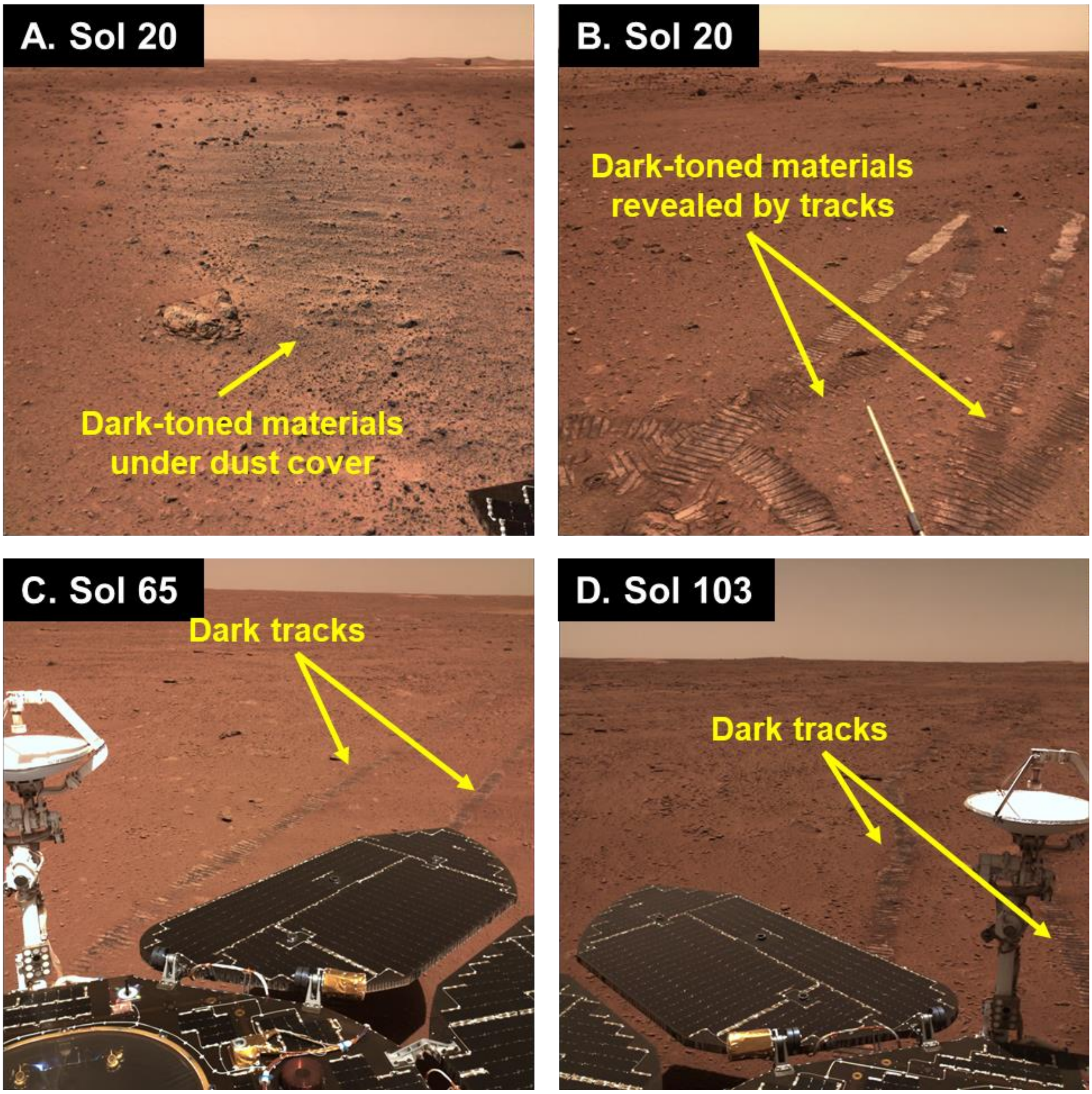

Supplementary Figure 1

Dark-toned materials underneath the dusty surface were revealed by the lander plume and rover tracks. The sol numbers indicate the sol when the NaTeCam images were taken. (A) Black-toned rocks and clasts underneath the dust cover were revealed by the descending plumes. (B-D) Rover tracks revealed dark-toned materials under the surface dust and soils on sols 20, 65, and 103, respectively. The NaTeCam image IDs are tabulated in **Supplementary Table 2.**

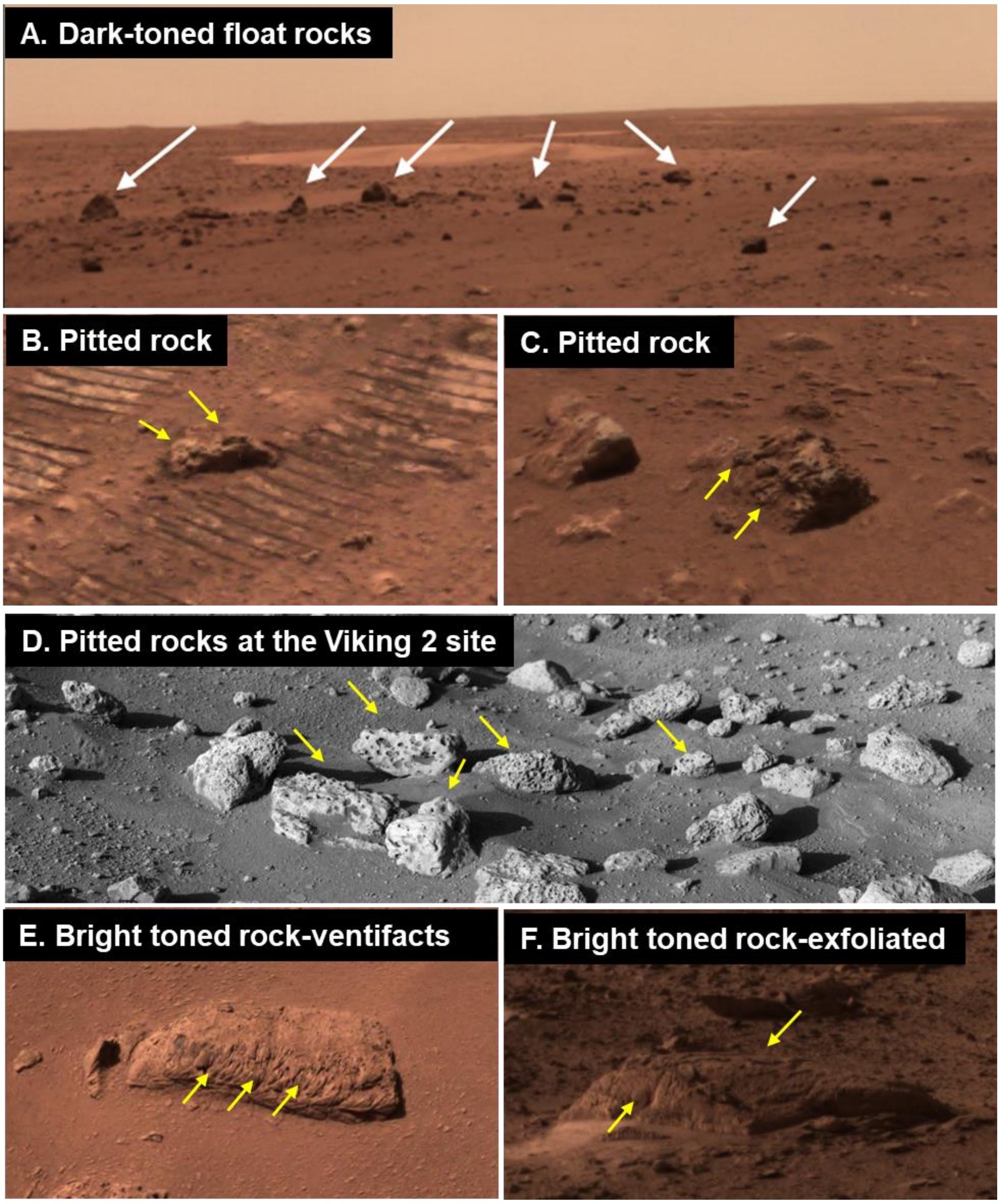

Supplementary Figure 2

Rocks observed at the Zhurong landing site. (A) dark-toned angular rocks as float rocks in the far ground, indicated by white arrows. Many were sitting close to the rim of impact craters. (B-C) Pitted rocks show sub-angular or sub-rounded shapes with pits (indicated by yellow arrows) similar to those observed at the Viking landing sites. (D) Pitted rocks were observed at the Viking 2 landing site (indicated by the arrows). (E-F) Bright-toned rocks show reddish-brown on the surface and dark-toned on the interior. Sub-angular or sub-rounded in morphology and partially buried in the regolith. The arrows indicate ventifact (E) and exfoliated (F) on the rock surface, suggesting that these rocks are subject to long-term aeolian reworks. The NaTeCam image IDs and Viking 2 images are listed in **Supplementary Table 2.**

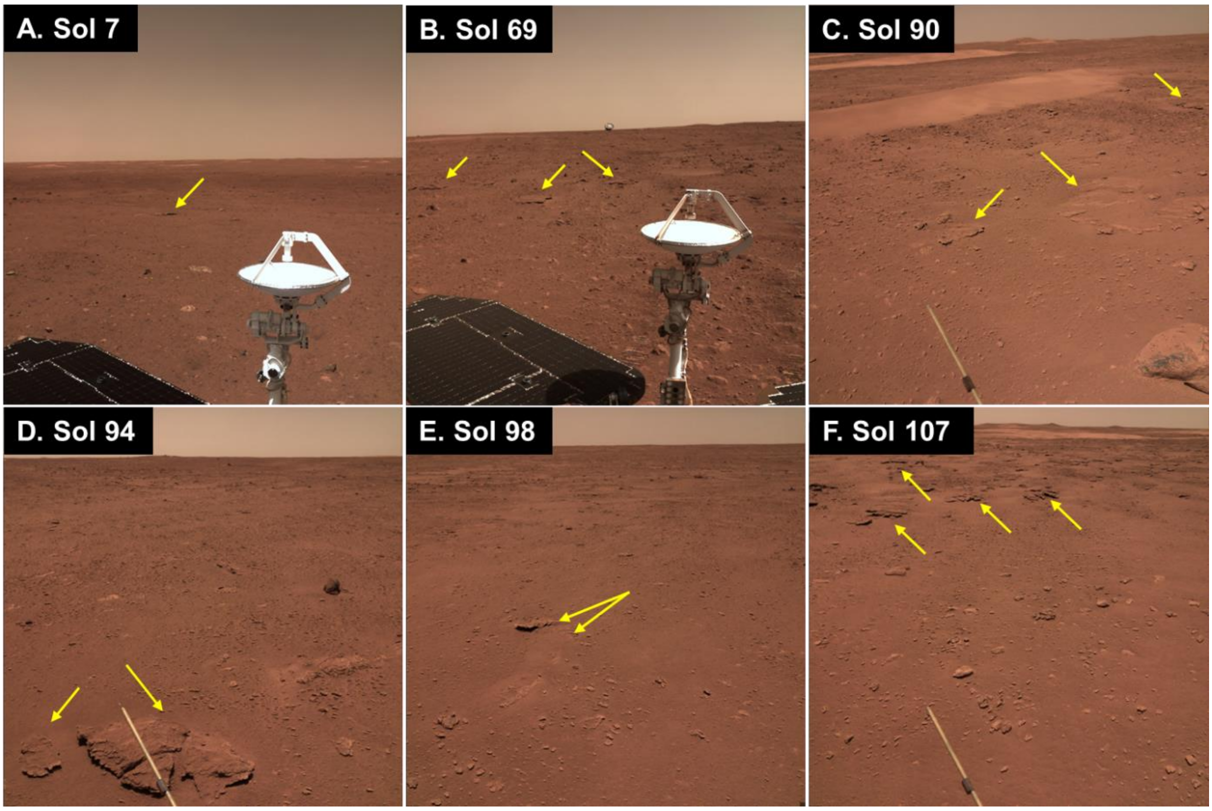

Supplementary Figure 3

Lithified duricrusts are present from the landing location to the later traverse. (A-C) Lithified duricrusts present as low-relief pavers in the regolith or as thin slabs partially buried in the regolith. The exposed slabs are likely formed due to differential weathering. (D) Fragments of the lithified duricrust show wavy edges and fine laminae. (E) A thin slab on the surface and concealed rocks with larger volumes buried in the subsurface. The slab and buried portion are of the same rock. (F) Low relief paver slabs are massively present in the later traverse. The NaTeCam image IDs are tabulated in **Supplementary Table 2.**

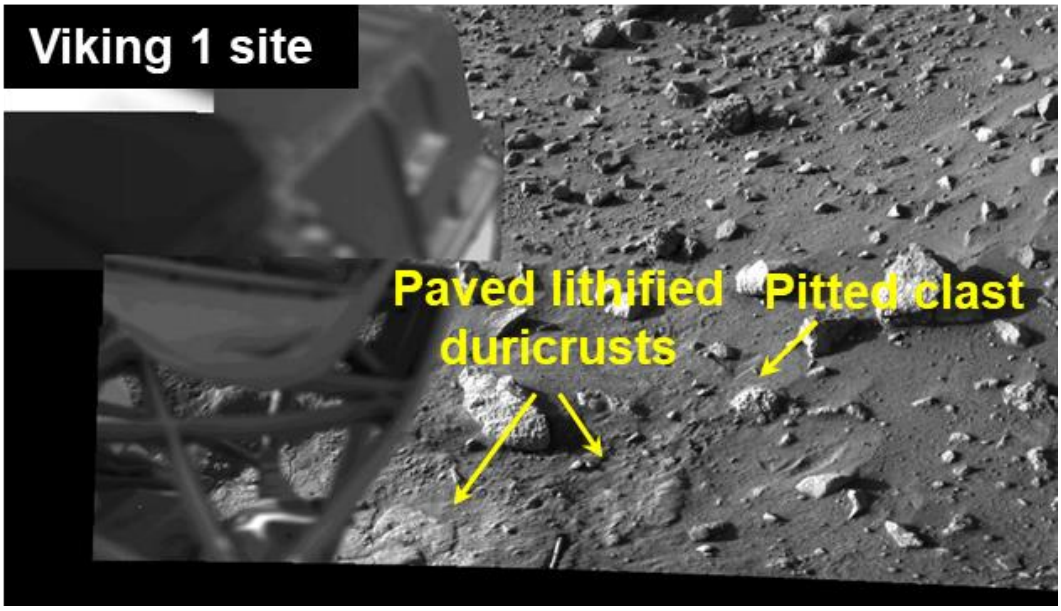

Supplementary Figure 4

The inferred lithified duricrusts and pitted rock clasts at the Viking 1 landing site (image source: PIA03164).

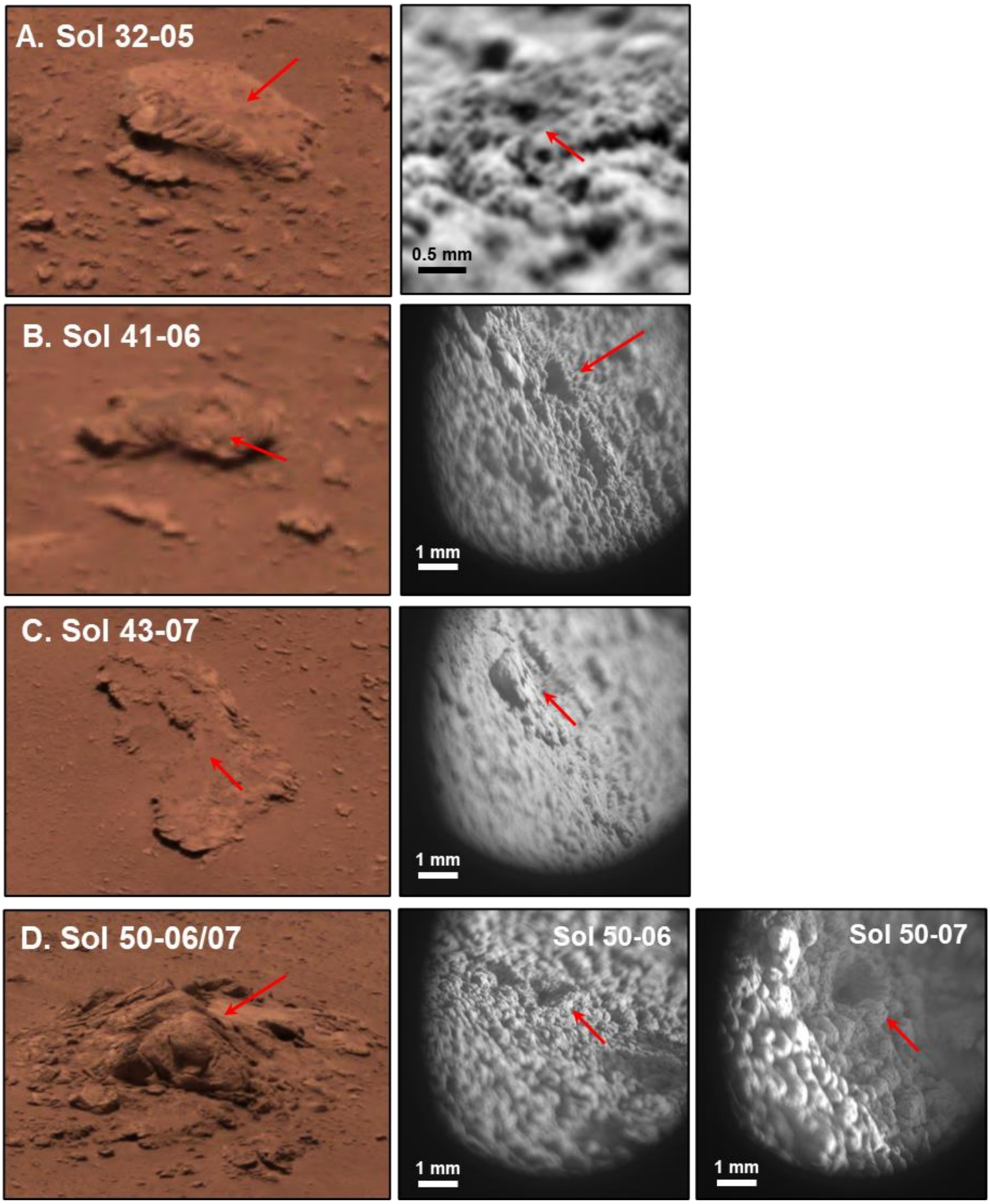

Supplementary Figure 5

The LIBS targets of nominal rocks but actually ablated soil and dust aggregates on the rock surface. The red arrows indicate the location of laser ablation in the NaTeCam images and micro-images. The IDs of the NaTeCam and TMI images are tabulated in **Supplementary Table 2.**

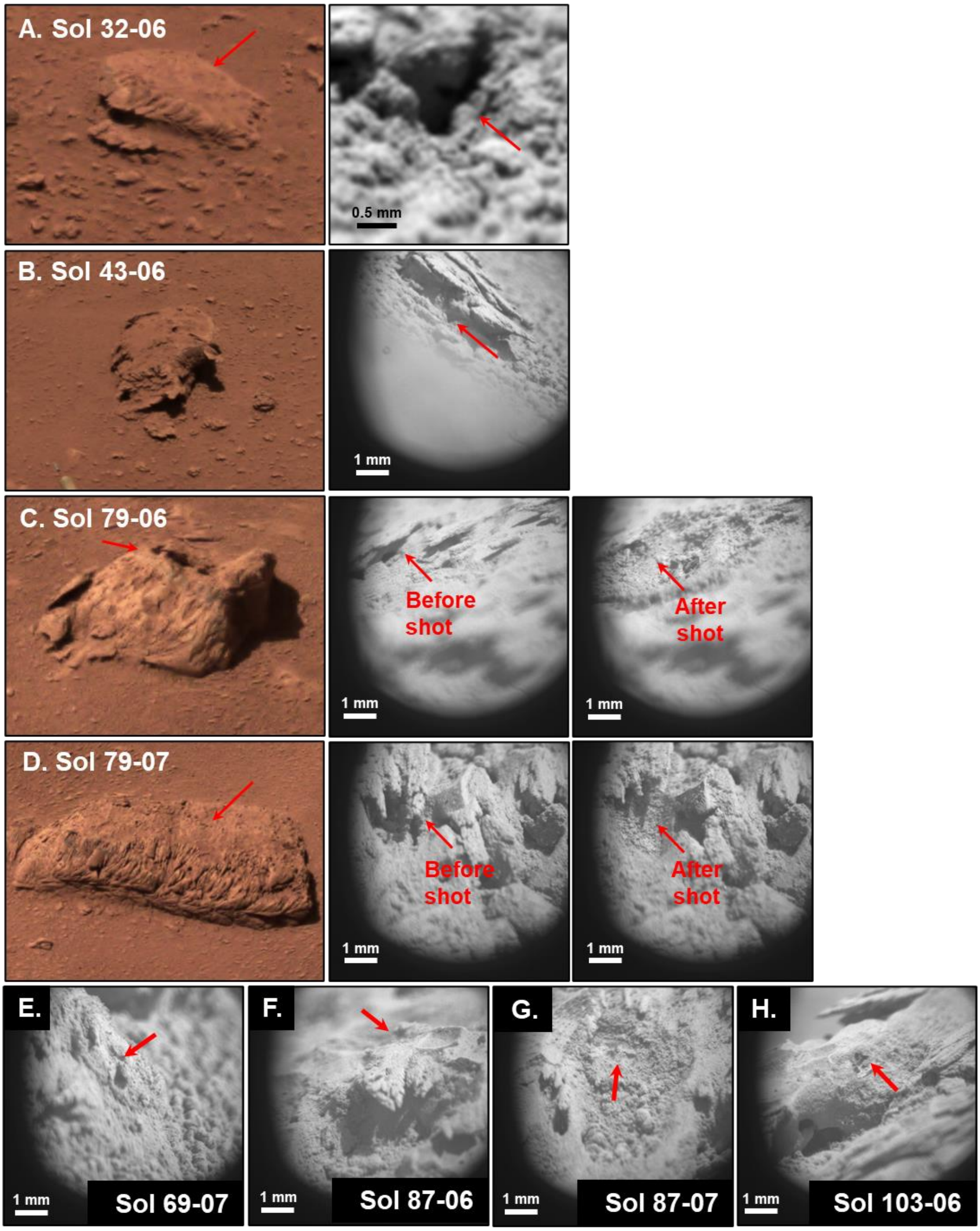

Supplementary Figure 6

Micro-images of cemented duricrusts coating on the rock surface. (A-D) Both NaTeCam images and micro-images were available for the targets. (E-H) Only micro-images after LIBS laser shot were available. The duricrusts show columnar or laminar micro-textures and are consistently shattered after laser shots, indicating a friable nature. The IDs of the NaTeCam and TMI images are tabulated in **Supplementary Table 2.**

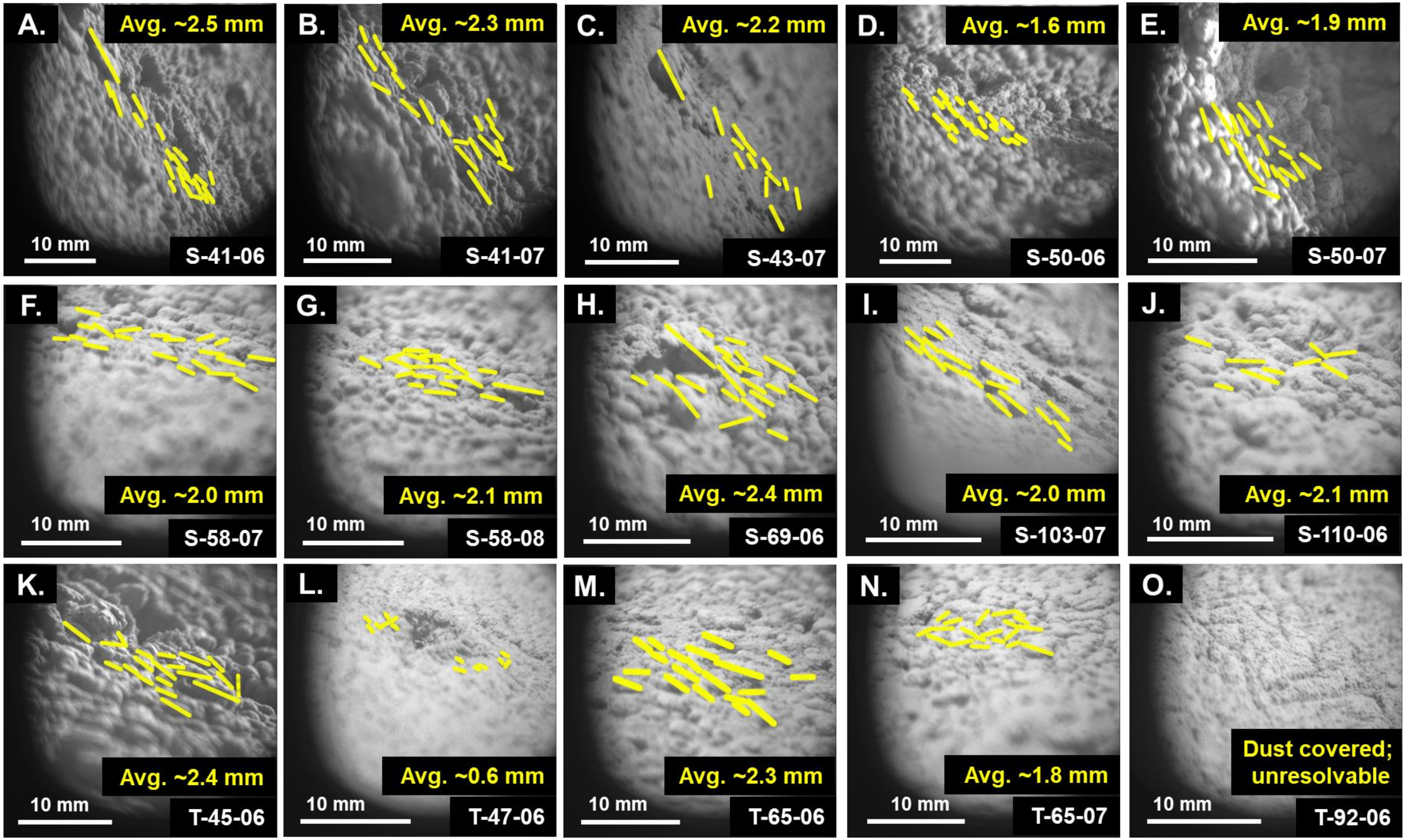
A.
Avg. ~2.5 mm
10 mm
S-41-06
B.
Avg. ~2.3 mm
10 mm
S-41-07
C.
Avg. ~2.2 mm
10 mm
S-43-07
D.
Avg. ~1.6 mm
10 mm
S-50-06
E.
Avg. ~1.9 mm
10 mm
S-50-07
F.
Avg. ~2.0 mm
10 mm
S-58-07
G.
Avg. ~2.1 mm
10 mm
S-58-08
H.
Avg. ~2.4 mm
10 mm
S-69-06
I.
Avg. ~2.0 mm
10 mm
S-103-07
J.
Avg. ~2.1 mm
10 mm
S-110-06
K.
Avg. ~2.4 mm
10 mm
T-45-06
L.
Avg. ~0.6 mm
10 mm
T-47-06
M.
Avg. ~2.3 mm
10 mm
T-65-06
N.
Avg. ~1.8 mm
10 mm
T-65-07
O.
Dust covered; unresolvable
10 mm
T-92-06

Supplementary Figure 7

Estimation of grain sizes of soil and sands using the micro-images obtained by the MarSCoDe. The average grain size of the large size portion is noted in each image. Fine portions, such as dust, cannot be resolved and were constrained to <0.2 mm based on the resolution limit of the micro-imaging system. The IDs of TMI images are tabulated in **Supplementary Table 2**.

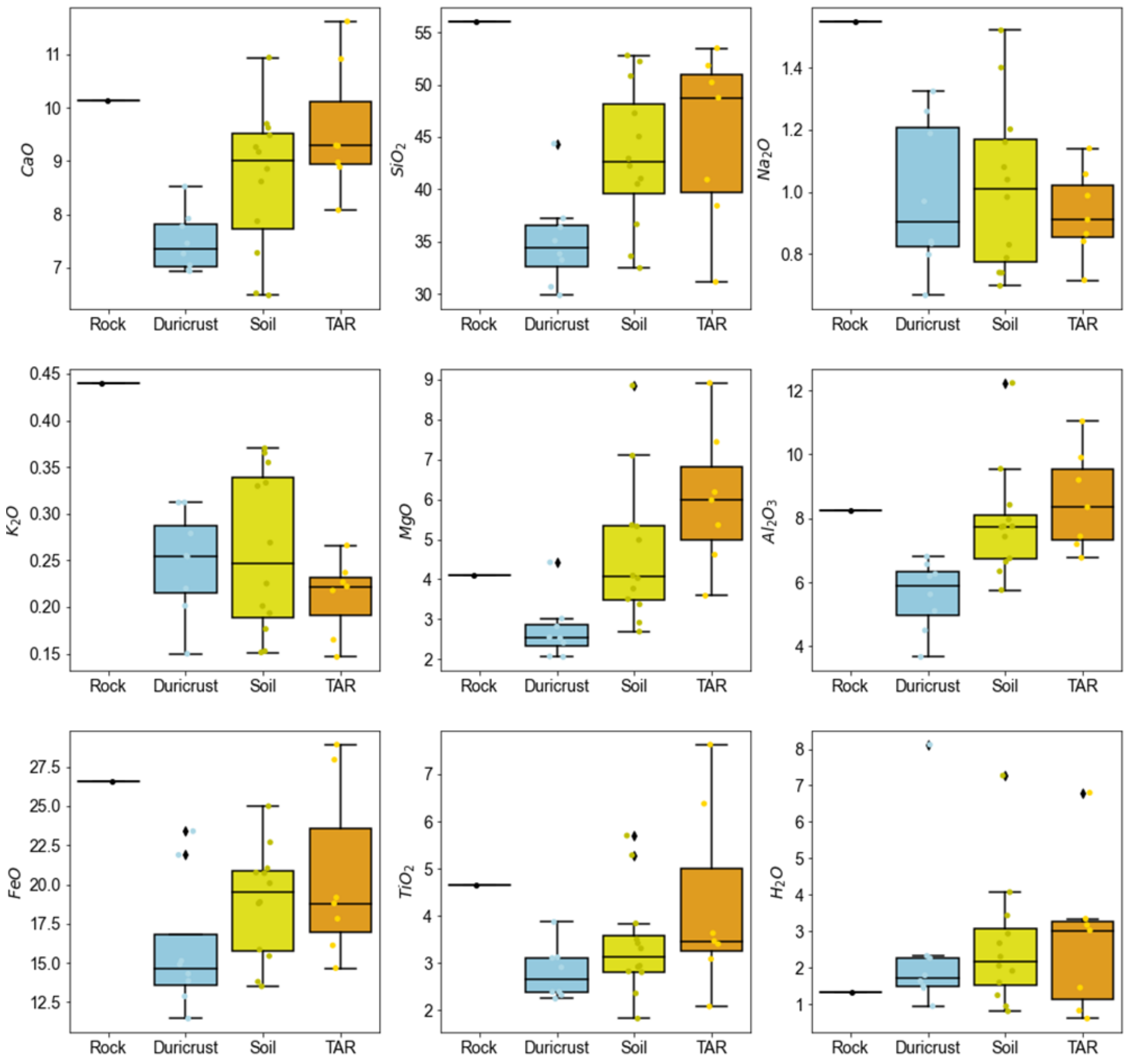

Supplementary Figure 8

Box and whisker plots of the composition for the measured igneous rock, duricrusts (combined lithified and cemented), soils and TAR sands. The center horizontal line represents median values for each category. Boxes show the range between the first and third quartiles. Whiskers represent 1.5 times the interquartile range (IQR) extending from the boxes, with diamonds showing the outliers beyond. Data points of each group are also plotted on top of the box and whiskers.

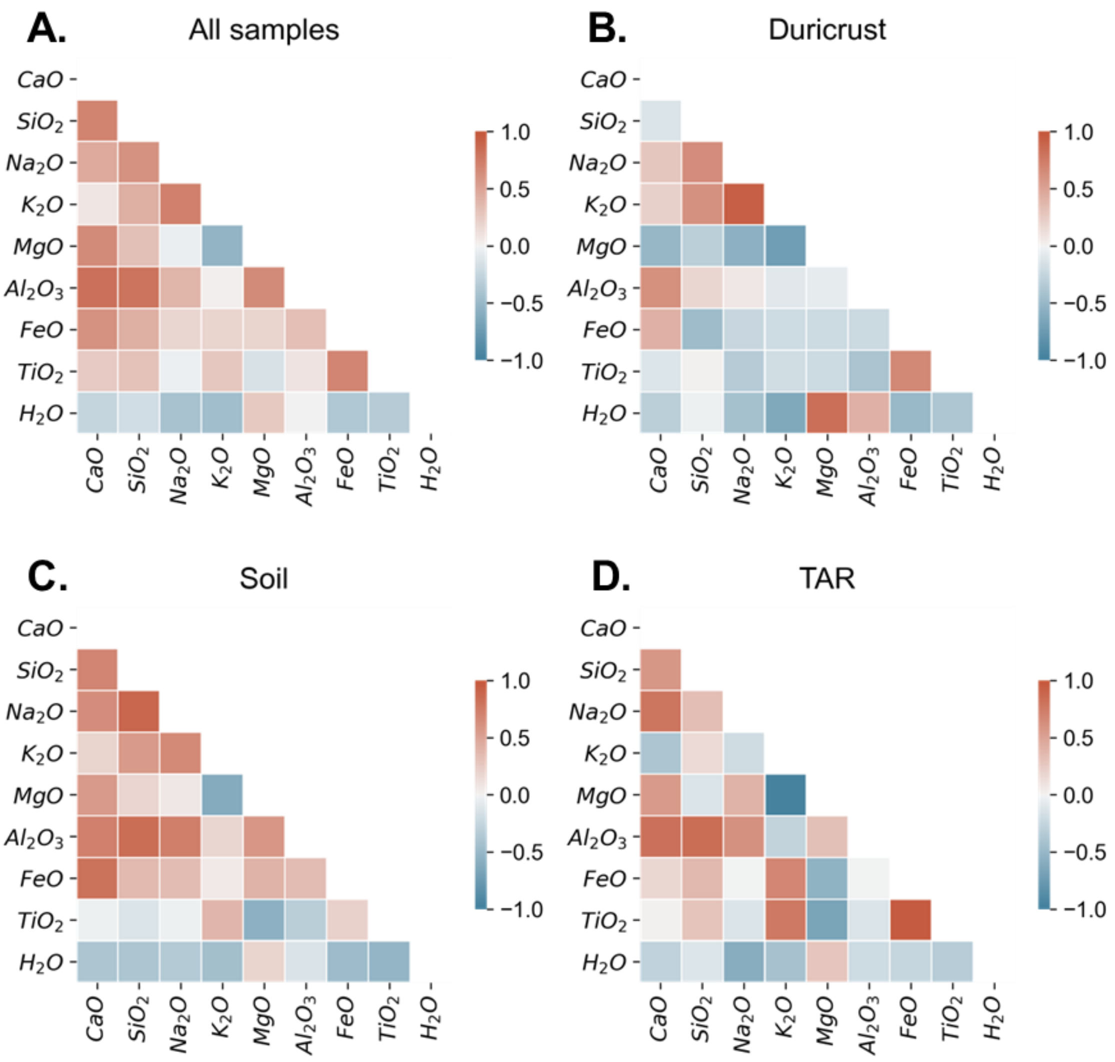

Supplementary Figure 9

Correlation matrix of LIBS measurements for (A) all samples, (B) duricrusts (combined lithified and cemented), (C) soils and (D) TAR sands. The color of each box represents the Pearson standard correlation coefficient between two variables. Red indicates a positive correlation, and blue indicates a negative correlation.

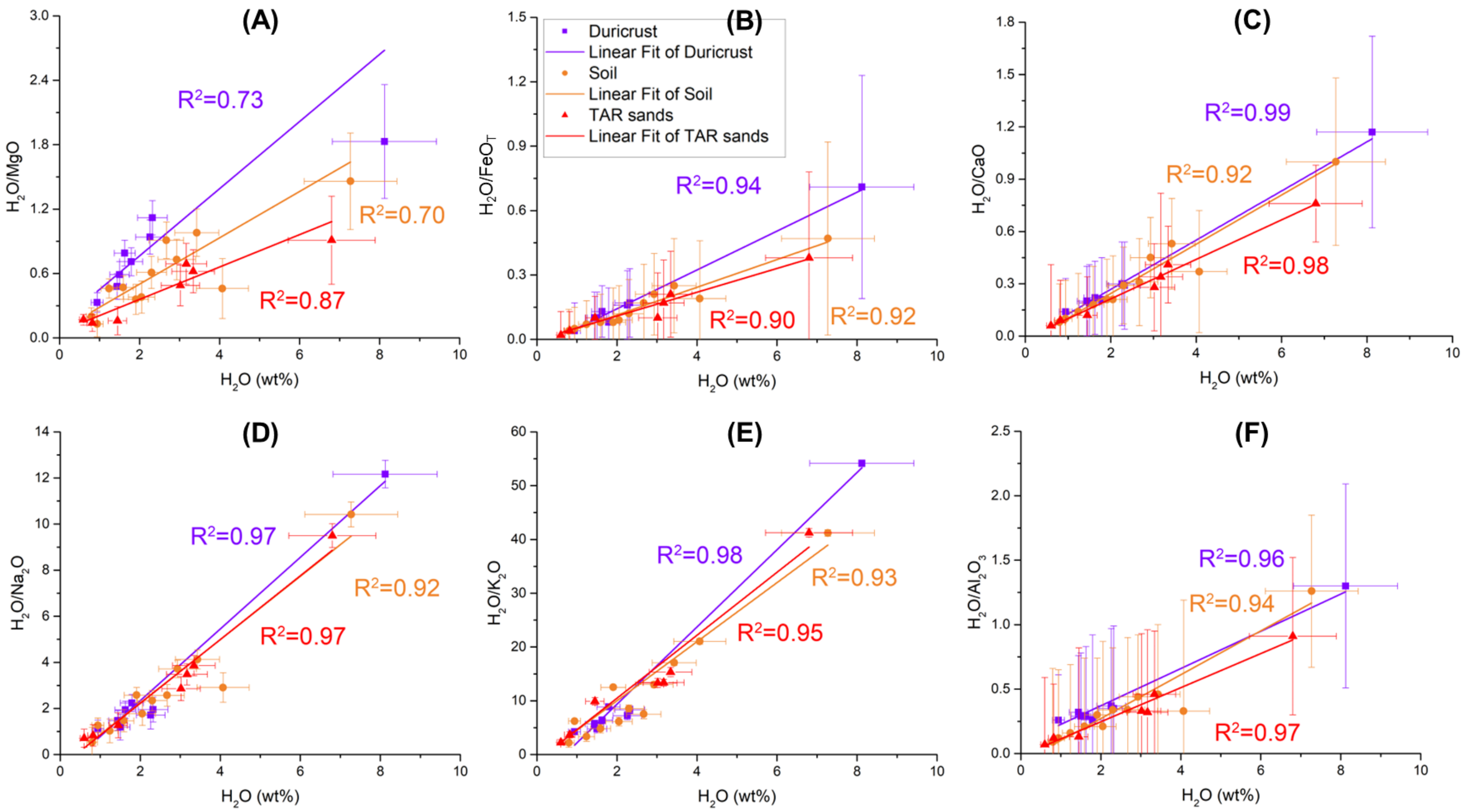

Supplementary Figure 10

Correlations suggest that the H_2O contents are independently present in the samples rather than being controlled by cation oxides. Correlations between H_2O contents versus ratios of (A) H_2O/MgO, (B) H_2O/FeO_T, (C) H_2O/CaO, (D) H_2O/Na_2O, (E) H_2O/K_2O, and (F) H_2O/Al_2O_3 in the categories of "duricrusts" (combined both lithified and cemented), "soils", and "TAR sands". Due to the low sample numbers in each category, we define $0.70 \leq R_2 < 0.90$ as a moderate correlation and $R^2 \geq 0.90$ as a strong correlation. Strong or moderate correlations are present in almost all sample categories.

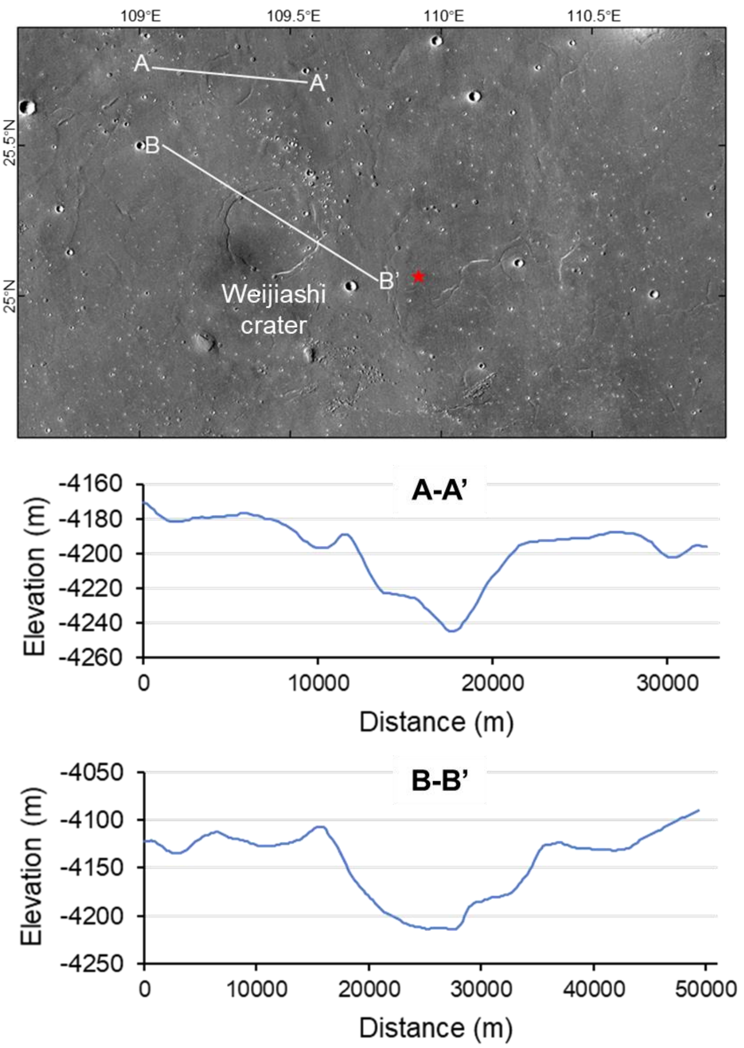

Supplementary Figure 11

Constraints on the depositional depth in the Zhurong landing site using two ghost (infilled) craters. An unnamed impact crater (25.703°N, 109.31°E; A-A') and the Weijiashi crater (25.22°N, 109.442°E; B-B') were used. The location of the Tianwen-1 lander is marked by the red star on the CTX mosaic image. The MOLA topographic profiles obtained crosslines A-A' and B-B', which were more than three times the crater diameters. With the known fresh impact crater geometry acquired [9] in the Utopia Planitia and the rim height measured by the MOLA DEM, the upper limit of the thickness of the overlying basal materials is estimated to be 395 m. This estimated result is consistent with previous estimations using similar methods in a larger region [10,11]. The CTX mosaic image is sourced from MurrayLab_CTX-Mosaic_beta01_E108_N24 (http://murray-lab.caltech.edu/CTX/tiles/beta01/E-108/).

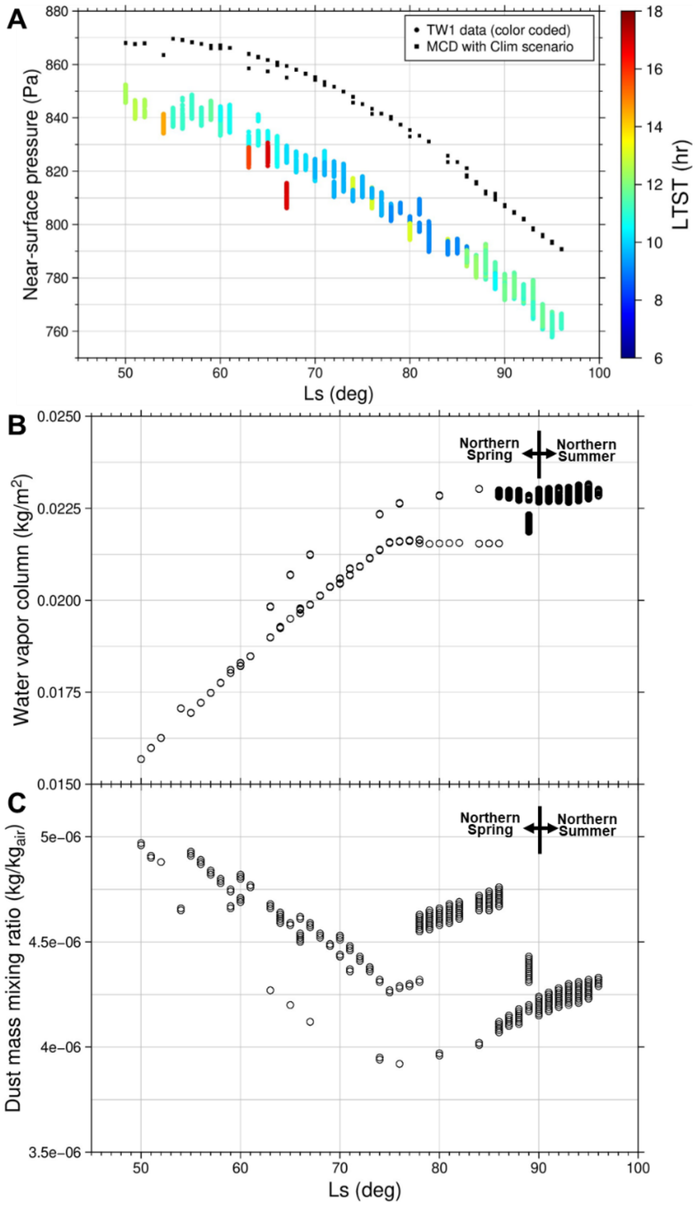

Supplementary Figure 12

Comparison of the measured meteorological data by the MCS to the numerical simulation results using the MCD. (A) Air pressure near the ground. The simulated results are in good agreement with the measured data, and both decrease due to seasonal changes. The colored bars represent the measured data, and solid black squares represent the simulation results at the same time and same location. The length of the colored bars represents the values of the parameters, and the color indicates the measurement time at the local true solar time (LTST). Note that the MCS only samples the local climate once a sol for ~5-50 min in a day time, which cannot represent the diurnal climate variations or the average values of a given sol. (B) and (C) show the simulated water vapor and dust mass in the air as seasonal changes. Substantial differences are present as the season turns from northern spring to summer.

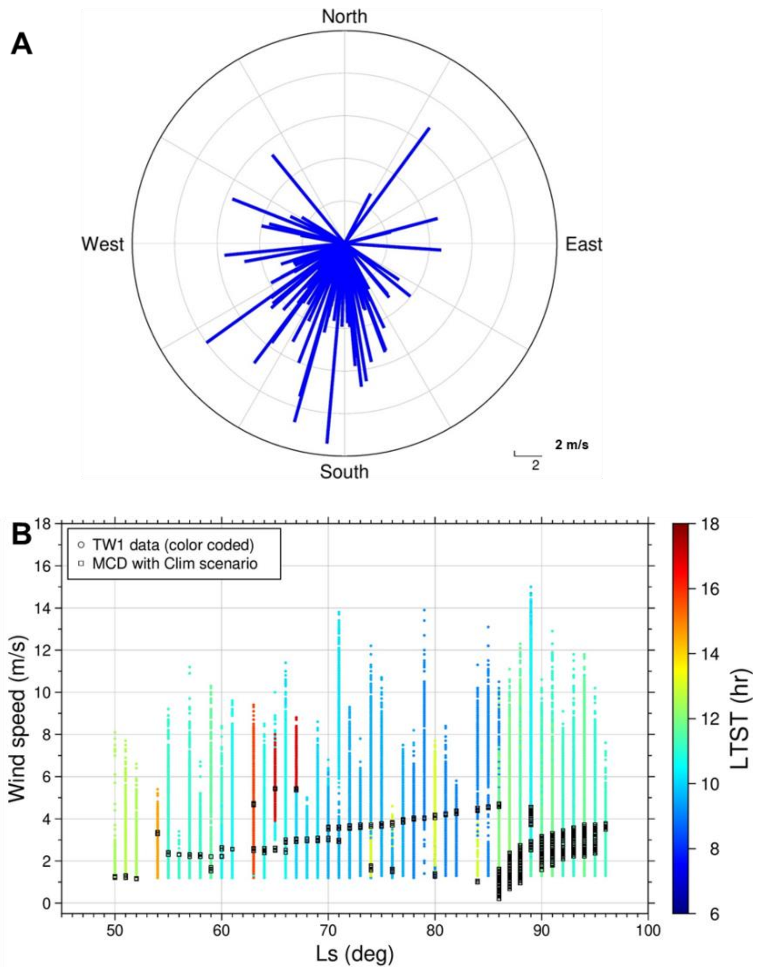

Supplementary Figure 13

Comparison of the measured wind data by the MCS to the numerical simulation results using the MCD. (A) A rose pattern of the direction and speed of the local wind along the Zhurong traverse recorded by the MCS up to sols 110 (equivalent to Ls 50°-100°). An average value of each sol is plotted, and the wind speed scale of 2 m/s is shown in the legend. The measurements were conducted during local daytime (06:00-18:00) and lasted for 5 to 50 minutes. (B) Measured local wind speed versus numerical simulation results. The colored bars represent the measured data, and solid black squares represent the simulation results at the same time and same location. The length of the colored bars represents the values of the parameters, and the color indicates the measurement time at the local true solar time (LTST). The measured wind speed shows a large variation during the daytime, while the simulated results demonstrate a similar trend but less perturbation.

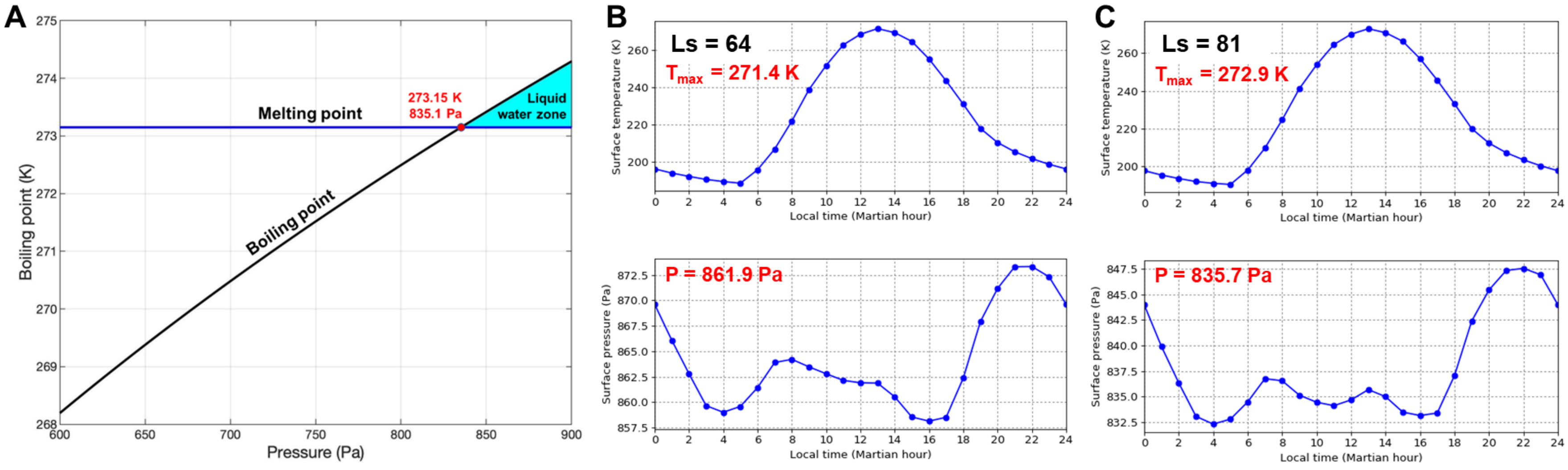

s

Supplementary Figure 14

A comparison of the simulated diurnal *T-P* variations in selected Ls at the Zhurong site with the conditions allow for liquid water to be present on the Martian surface. (A) The water melting line (blue line) is independent of the pressure, and the water boiling curve (black curve) increases with increasing air pressure. The intersection (273.15 K and 835.1 Pa) of the melting and boiling curves forms the liquid water zone (in teal), within which liquid water is present (i.e., melting but not boiling). (B) The simulated diurnal *T-P* variations of Ls = 64°, of which the highest diurnal T is 271.4 K, and the corresponding *P* is 861.9 Pa. (C) The simulated diurnal T-P variations of Ls = 81°, of which the highest diurnal T is 272.9 K, and the corresponding *P* is 835.7 Pa. While the pressure conditions meet the threshold for liquid water (835.1 Pa), the simulated *T* is slightly lower but close to the threshold (273.15 K). Considering that the MCD simulations have an error of ± 10 K, transient liquid water would likely be present at the Zhurong site around the northern summer solstice. If hydrated salts present in the topsoil are involved and depressed the eutectic points, transient brine may be present for a short time diurnally during the warmest season.

（a）

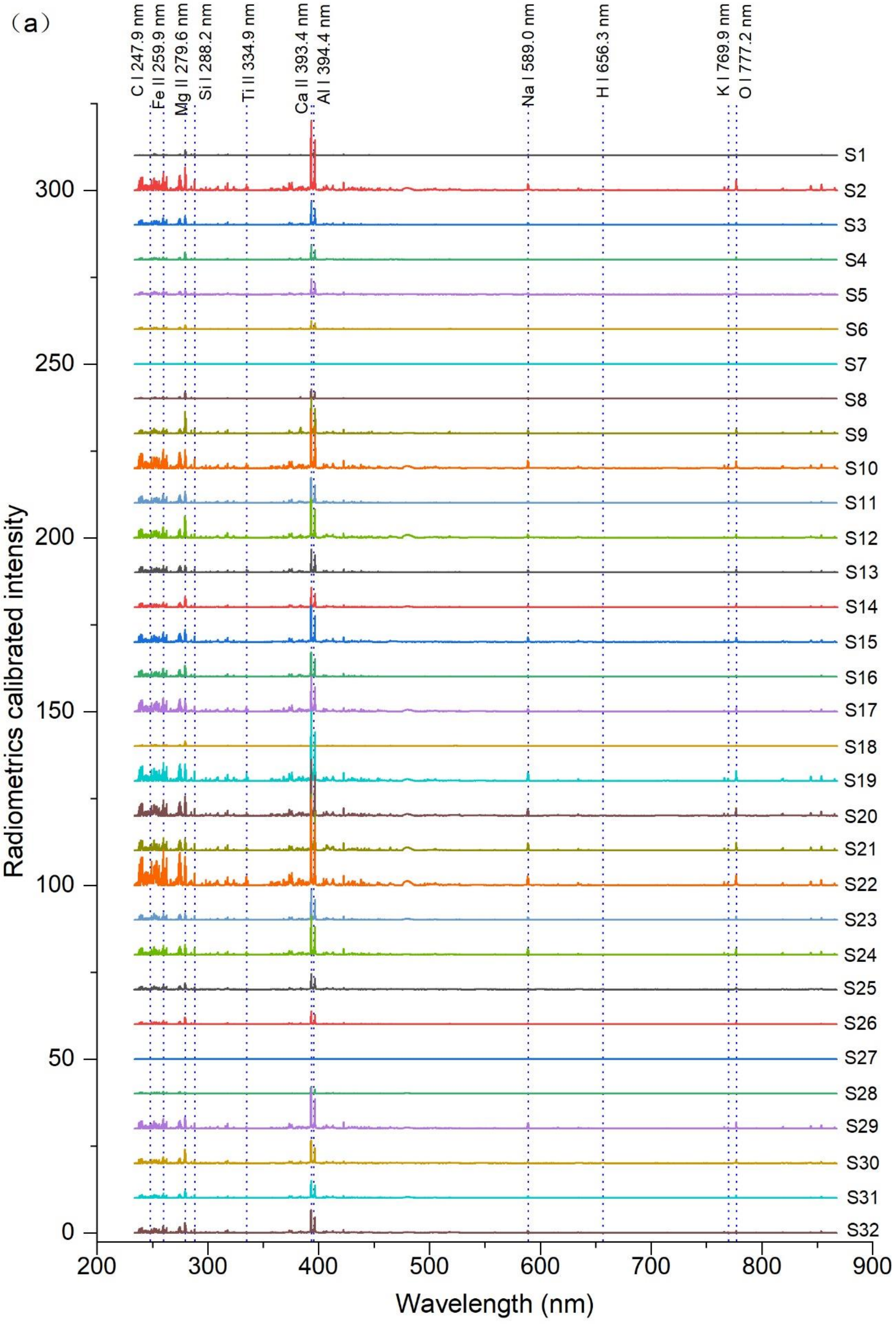
C I 247.9 nm
Fe II 259.9 nm
Mg II 279.6 nm
Si I 288.2 nm
Ti II 334.9 nm
Ca II 393.4 nm
Al I 394.4 nm
Na I 589.0 nm
H I 656.3 nm
K I 769.9 nm
O I 777.2 nm
S1
S2
S3
S4
S5
S6
S7
S8
S9
S10
S11
S12
S13
S14
S15
S16
S17
S18
S19
S20
S21
S22
S23
S24
S25
S26
S27
S28
S29
S30
S31
S32
Radiometrics calibrated intensity
0
50
100
150
200
250
300
200
300
400
500
600
700
800
900
Wavelength (nm)

（b）
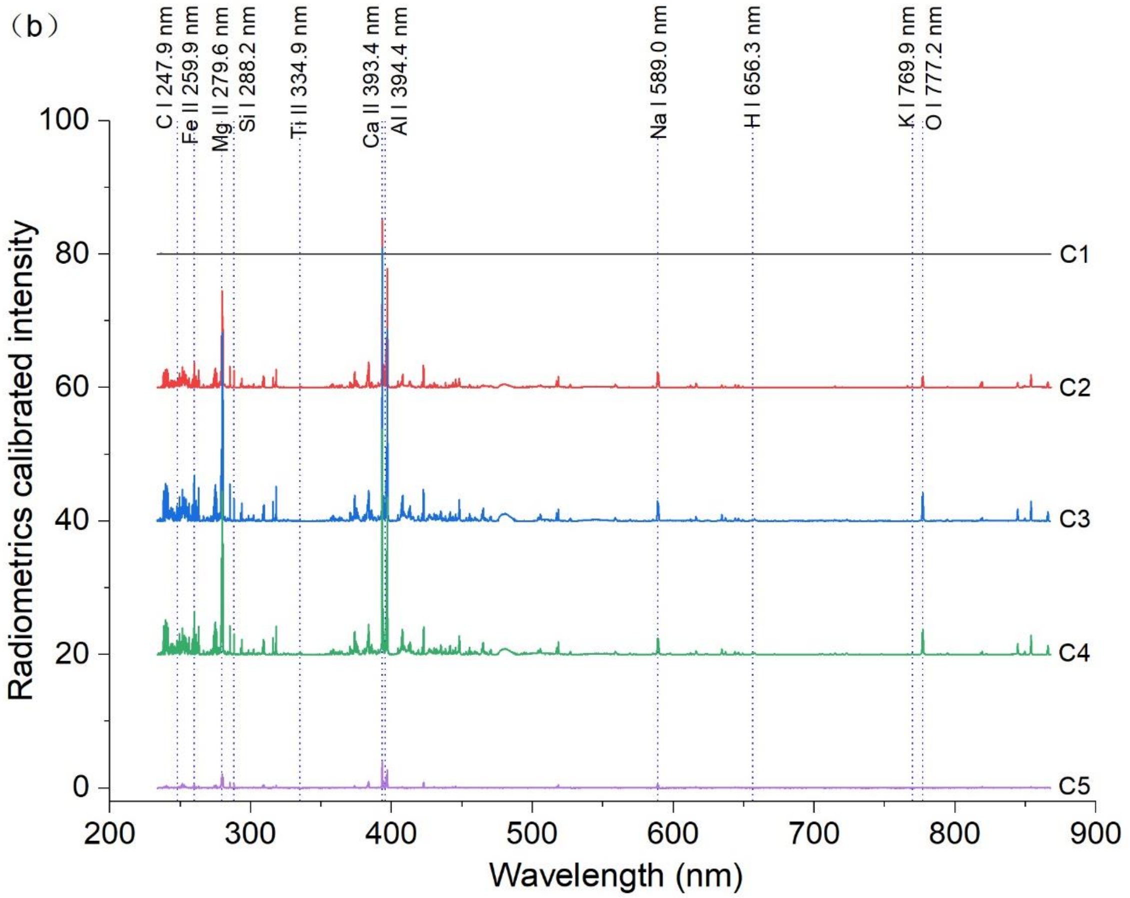
C I 247.9 nm
Fe II 259.9 nm
Mg II 279.6 nm
Si I 288.2 nm
Ti II 334.9 nm
Ca II 393.4 nm
Al I 394.4 nm
Na I 589.0 nm
H I 656.3 nm
K I 769.9 nm
O I 777.2 nm
C1
C2
C3
C4
C5
Radiometrics calibrated intensity
Wavelength (nm)

（c）
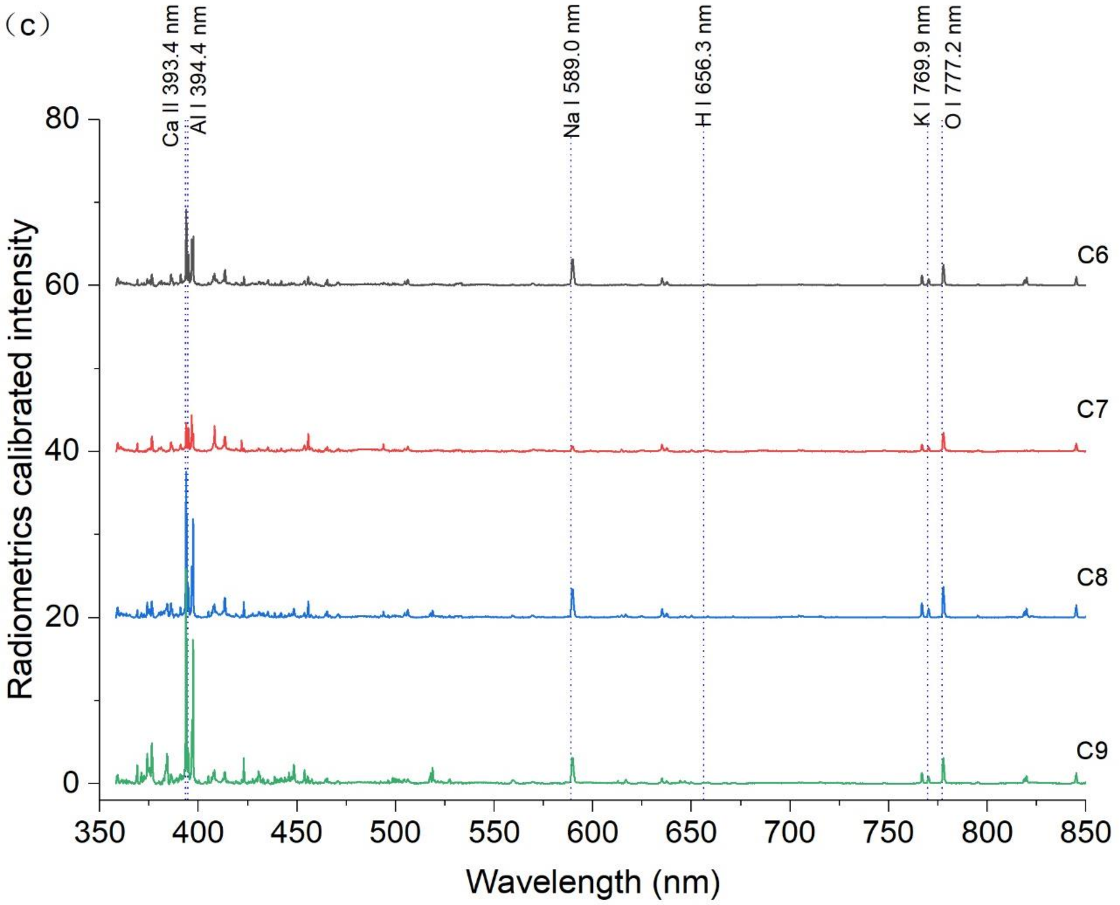
Ca II 393.4 nm
Al I 394.4 nm
Na I 589.0 nm
H I 656.3 nm
K I 769.9 nm
O I 777.2 nm
C6
C7
C8
C9
Radiometrics calibrated intensity
Wavelength (nm)

Supplementary Figure 15

Preprocessed raw LIBS spectra further treated and analyzed in this work. (a) Thirty-two spectra of 32 scientifical targets measured during the first 110 sols; (b) five spectra of the Norite onboard calibration target recorded during the same period; (c) four spectra of the 4 prepared igneous reference samples acquired in a Mars simulation chamber and with the replicate of the MarSCoDe instrument.

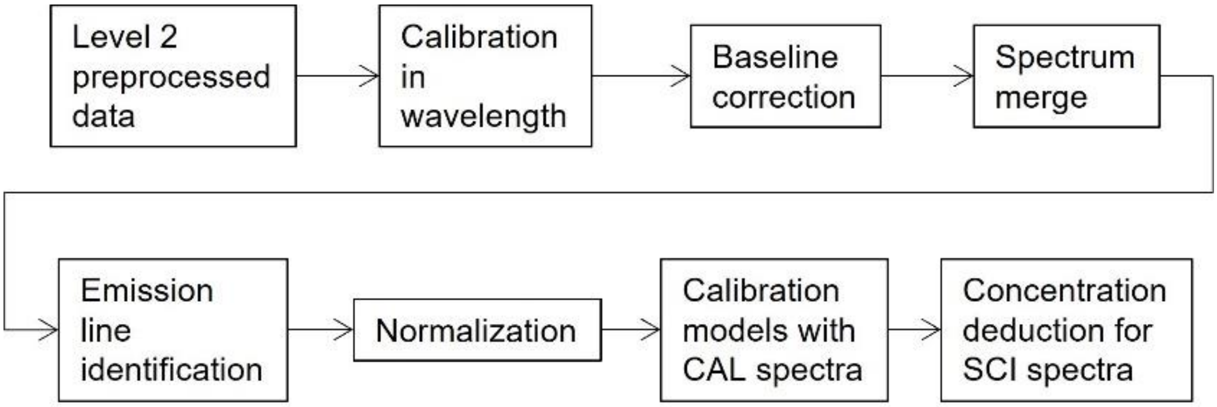

Supplementary Figure 16

The flowchart of the LIBS data treatment method used in this work.

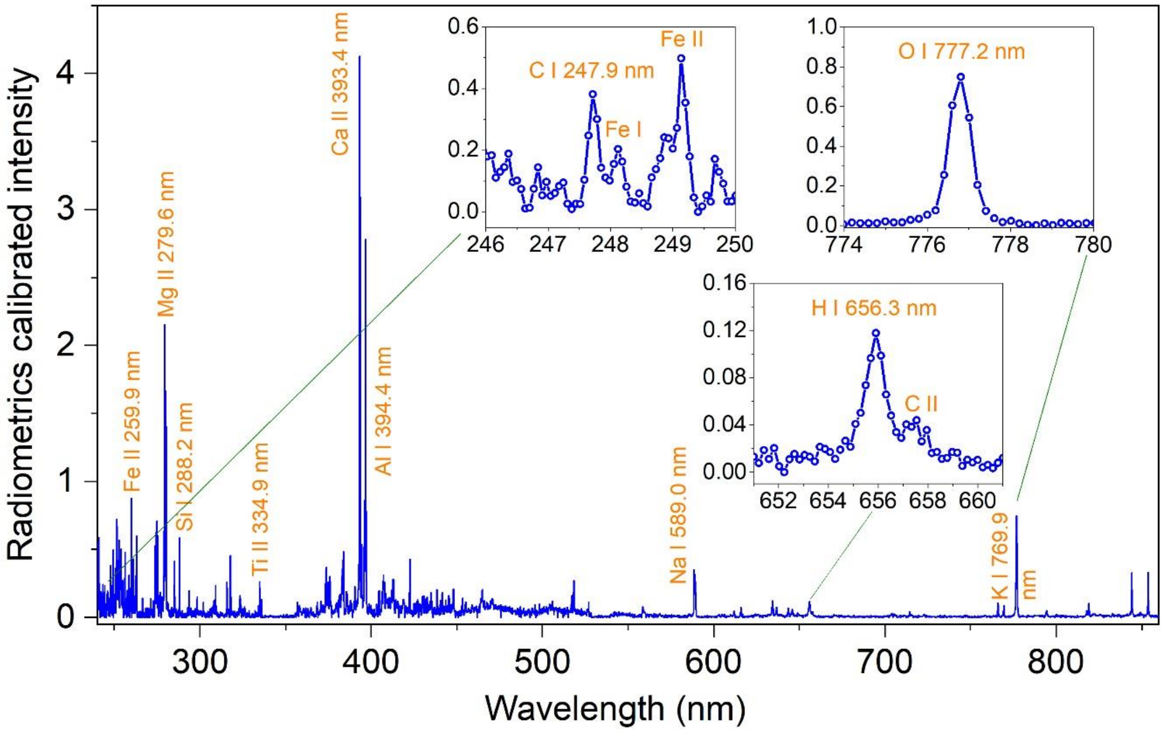

Supplementary Figure 17

A typical merged baseline-corrected spectrum of a scientific target together with the indications of the principal lines of the elements considered in this work. The target is D 32-06, and the spectrum ID is S4 (HX1-Ro_GRAS_MarSCoDe-LIBS-I-06_SCI_N_20210615174910_20210615174931_00032_A.2B).

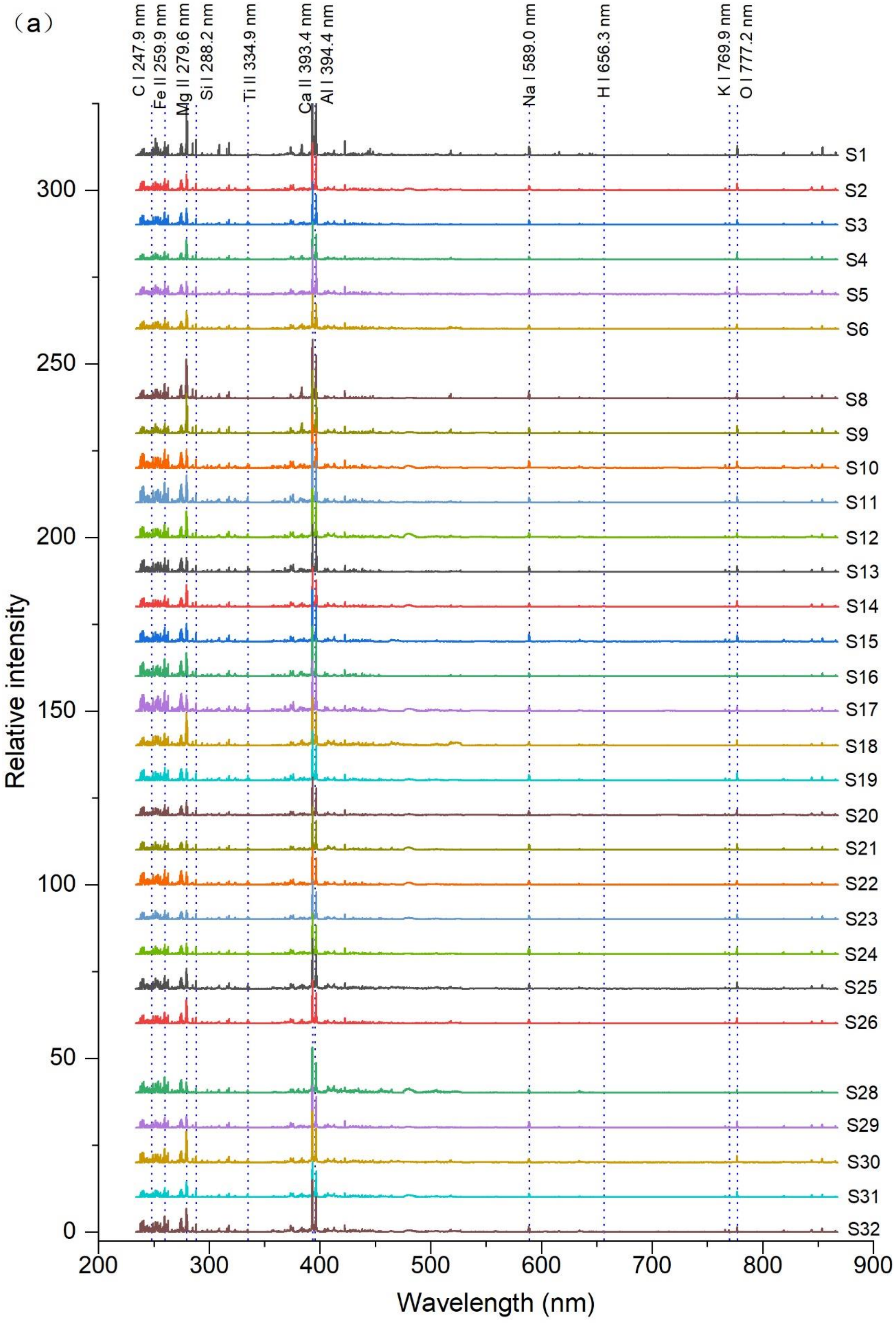
（a）
C I 247.9 nm
Fe II 259.9 nm
Mg II 279.6 nm
Si I 288.2 nm
Ti II 334.9 nm
Ca II 393.4 nm
Al I 394.4 nm
Na I 589.0 nm
H I 656.3 nm
K I 769.9 nm
O I 777.2 nm
S1
S2
S3
S4
S5
S6
S8
S9
S10
S11
S12
S13
S14
S15
S16
S17
S18
S19
S20
S21
S22
S23
S24
S25
S26
S28
S29
S30
S31
S32
Relative intensity
Wavelength (nm)
0
50
100
150
200
250
300
200
300
400
500
600
700
800
900

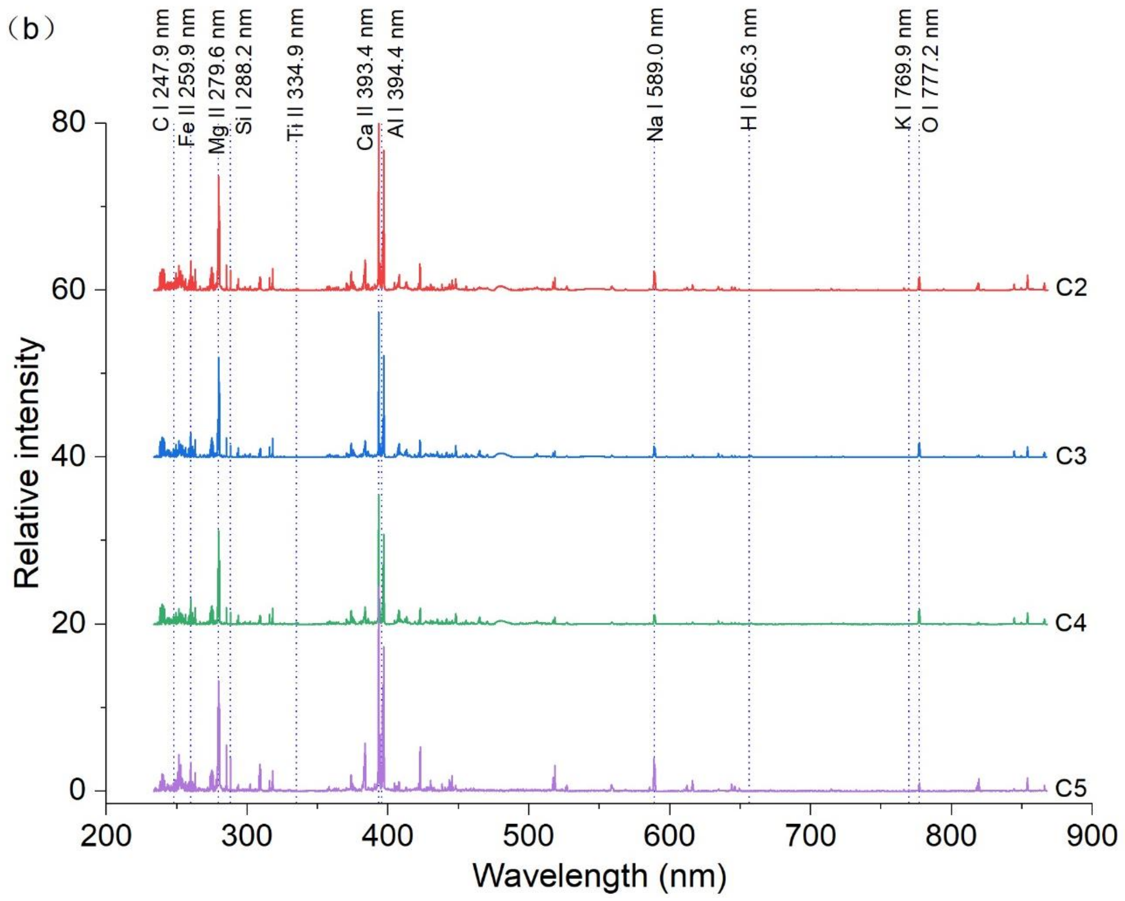

Supplementary Figure 18

Spectra normalized by C I 247.9 nm line. (a) Thirty valid spectra of 30 scientifical targets normalized by C I 247.9 nm line; (b) four valid spectra of the Norite onboard calibration target normalized by C I 247.9 nm line.

(a)

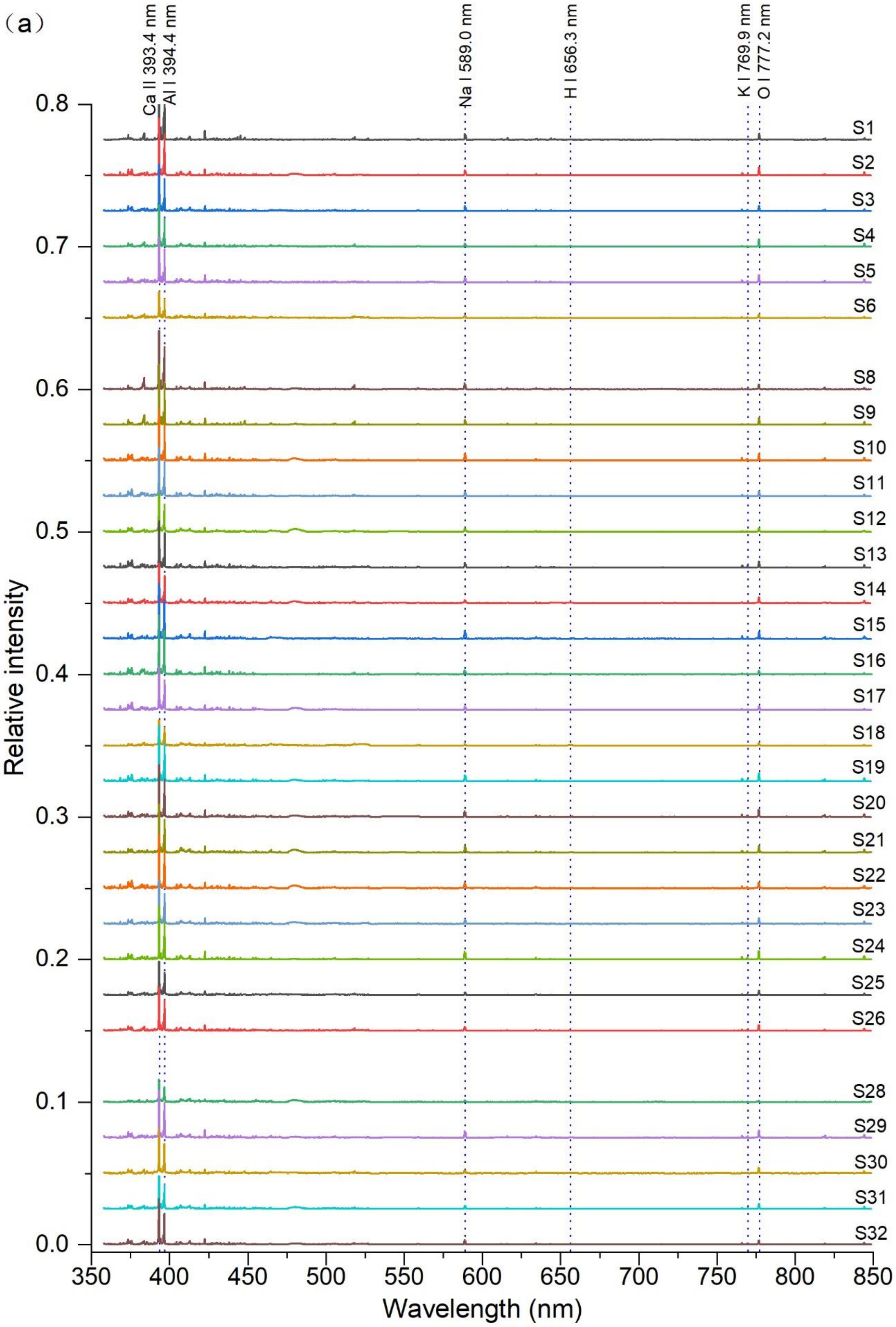

Ca II 393.4 nm
Al I 394.4 nm
Na I 589.0 nm
H I 656.3 nm
K I 769.9 nm
O I 777.2 nm
S1
S2
S3
S4
S5
S6
S8
S9
S10
S11
S12
S13
S14
S15
S16
S17
S18
S19
S20
S21
S22
S23
S24
S25
S26
S28
S29
S30
S31
S32
Relative intensity
0.0
0.1
0.2
0.3
0.4
0.5
0.6
0.7
0.8
Wavelength (nm)
350
400
450
500
550
600
650
700
750
800
850

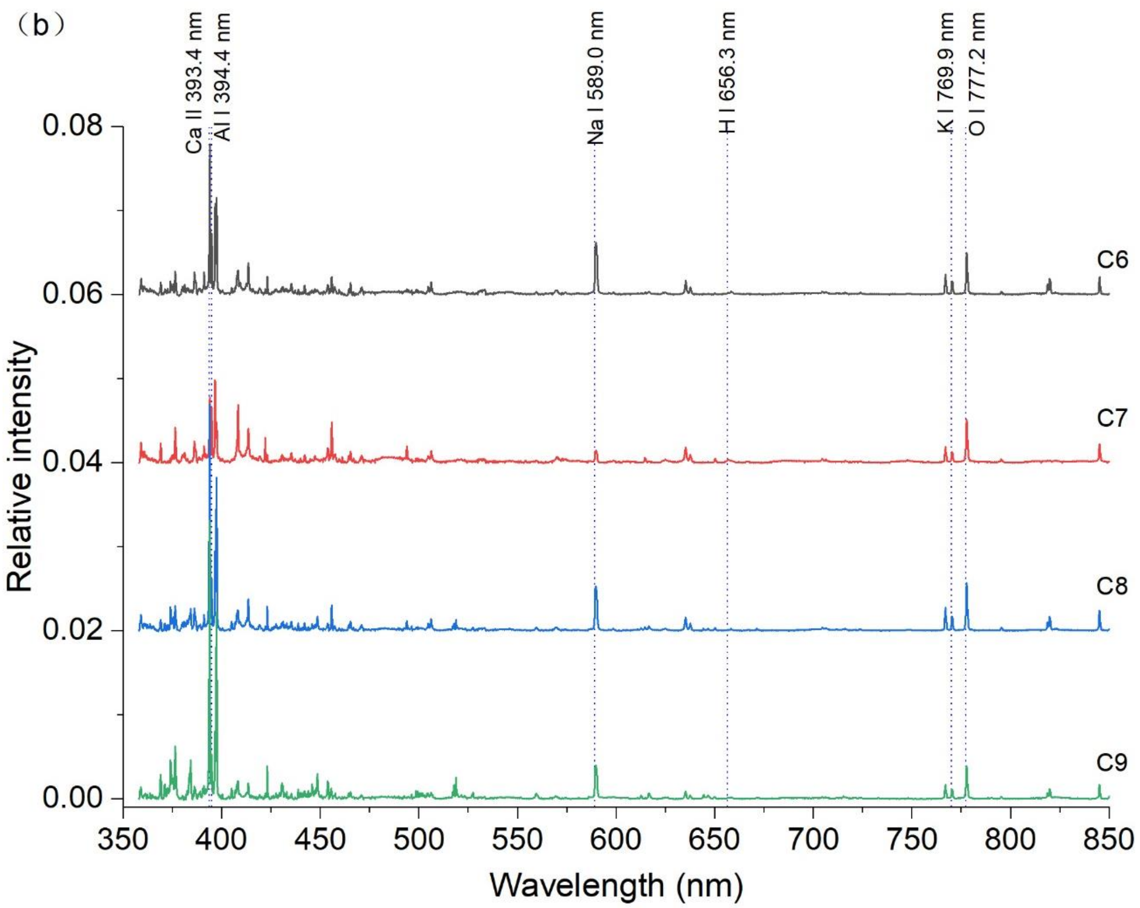

Supplementary Figure 19

Spectra normalized by total spectral intensity. (a) Thirty valid spectra of 30 scientifical targets normalized by total spectrum intensity, and presented in the spectral range from 350 nm to 850 nm; (b) four spectra of the four prepared igneous reference samples acquired in a Mars simulation chamber and with the replicate of the MarSCoDe instrument.

Supplementary Table 1. Major oxides and H_2O concentrations measured by MarSCoDe LIBS during the first 110 sols.

Igneous rocks											
**Sample ID**	**Descriptions**	**Distance**	**CaO**	**SiO_2**	**Na_2O**	**K_2O**	**MgO**	**Al_2O_3**	**FeO_T**	**TiO_2**	**H_2O**
R-45-07	The dark-toned rock; measured on rock material [HX1-Ro_GRAS_MarSCoDe-LIBS-I-07_SCI_N_20210629074321_20210629074342_00045_A.2B]	2.6978 m	10.13	56.04	1.55	0.44	4.10	8.24	26.55	4.65	1.32
Lithified duricrusts											
**Sample ID**	**Descriptions**	**Distance**	**CaO**	**SiO_2**	**Na_2O**	**K_2O**	**MgO**	**Al_2O_3**	**FeO_T**	**TiO_2**	**H_2O**
LD-100-07	The lithified duricrust; jagged patterns [HX1-Ro_GRAS_MarSCoDe-LIBS-I-07_SCI_N_20210824143501_20210824143522_00100_A.2B]	3.1479 m	8.52	29.86	0.80	0.20	2.53	6.80	23.40	3.10	1.79
Cemented duricrusts											
**Sample ID**	**Descriptions**	**Distance**	**CaO**	**SiO_2**	**Na_2O**	**K_2O**	**MgO**	**Al_2O_3**	**FeO_T**	**TiO_2**	**H_2O**
D-32-06	Cemented duricrust [HX1-Ro_GRAS_MarSCoDe-LIBS-I-06_SCI_N_20210615174910_20210615174931_00032_A.2B]	3.4132 m	6.94	33.82	0.67	0.15	4.43	6.26	11.45	2.38	8.12
D-69-07	Weak cement [HX1-Ro_GRAS_MarSCoDe-LIBS-I-07_SCI_N_20210723193446_20210723193507_00069_A.2B]	2.8879 m	7.05	30.68	0.97	0.25	3.02	4.50	15.13	2.32	1.44
D-79-06	Weak cement-layered [HX1-Ro_GRAS_MarSCoDe-LIBS-I-06_SCI_N_20210803015454_20210803015515_00079_A.2B]	1.9108 m	7.92	35.09	1.19	0.28	2.07	6.56	13.86	2.24	2.32
D-79-07	Weak cement [HX1-Ro_GRAS_MarSCoDe-LIBS-I-07_SCI_N_20210803020843_20210803020904_00079_A.2B]	1.8332 m	6.95	33.25	0.83	0.22	2.82	3.67	21.88	3.87	0.94
D-87-06	Weak cement [HX1-Ro_GRAS_MarSCoDe-LIBS-I-06_SCI_N_20210811071011_20210811071032_00087_A.2B]	2.3684 m	7.26	37.22	0.84	0.26	2.06	5.63	12.87	3.12	1.63
D-87-07	Weak cement [HX1-Ro_GRAS_MarSCoDe-LIBS-I-07_SCI_N_20210811072400_20210811072421_00087_A.2B]	3.4096 m	7.46	44.36	1.32	0.31	2.41	6.19	14.30	2.90	2.26
D-103-06	Weak cement-layered [HX1-Ro_GRAS_MarSCoDe-LIBS-I-06_SCI_N_20210827161232_20210827161253_00103_A.2B]	2.7767 m	7.78	36.37	1.26	0.31	2.55	5.11	14.91	2.39	1.50
		Duricrust average (wt%)	7.34	35.83	1.01	0.25	2.77	5.42	14.92	2.74	2.60
		SD (wt%)	0.37	4.01	0.23	0.05	0.75	0.98	3.08	0.55	2.30

Soils												
**Sample ID**	**Descriptions**	**Distance**	**CaO**	**SiO_2**	**Na_2O**	**K_2O**	**MgO**	**Al_2O_3**	**FeO_T**	**TiO_2**	**H_2O**
S-32-05	Soil cover on the rock target; grain size unresolvable [HX1-Ro_GRAS_MarSCoDe-LIBS-I-05_SCI_N_20210615173712_20210615173733_00032_A.2B]	3.5133 m	7.87	41.02	0.98	0.27	3.77	6.75	18.79	3.50	2.30
S-41-06	Soil cover on the rock target; mean grain size 2.5 mm [HX1-Ro_GRAS_MarSCoDe-LIBS-I-06_SCI_N_20210625034210_20210625034231_00041_A.2B]	4.2298 m	8.62	47.26	1.04	0.35	2.92	7.97	15.84	2.82	2.67
S-41-07	Soil cover on the rock target; mean grain size 2.3 mm [HX1-Ro_GRAS_MarSCoDe-LIBS-I-07_SCI_N_20210625035429_20210625035450_00041_A.2B]	3.3846 m	6.48	33.61	0.79	0.23	4.03	6.64	13.80	2.80	2.93
S-43-07	Soil cover on the rock target; mean grain size 2.2 mm [HX1-Ro_GRAS_MarSCoDe-LIBS-I-07_SCI_N_20210627000435_20210627000456_00043_A.2B]	3.1756 m	10.94	52.78	1.40	0.19	8.84	12.23	21.03	1.82	4.07
S-50-06	Soil cover on the rock target; mean grain size 1.5 mm [HX1-Ro_GRAS_MarSCoDe-LIBS-I-06_SCI_N_20210704104745_20210704104806_00050_A.2B]	4.1733 m	8.86	42.24	1.08	0.33	3.38	7.73	20.76	5.70	1.59
S-50-07	Soil cover on the rock target; dust rich; mean 1.9 mm [HX1-Ro_GRAS_MarSCoDe-LIBS-I-07_SCI_N_20210704110004_20210704110025_00050_A.2B]	2.7001 m	7.28	32.47	0.70	0.18	4.98	5.76	15.44	2.35	7.27
S-58-07	Soil sample; mean grain size 2.0 mm [HX1-Ro_GRAS_MarSCoDe-LIBS-I-07_SCI_N_20210712091310_20210712091331_00058_A.2B]	2.9216 m	9.62	52.21	1.52	0.37	4.09	8.42	20.73	2.94	0.80
S-58-08	Soil sample; mean grain size 2.1 mm [HX1-Ro_GRAS_MarSCoDe-LIBS-I-08_SCI_N_20210712092659_20210712092720_00058_A.2B]	2.9454 m	9.26	36.66	0.74	0.15	5.32	6.34	24.99	3.84	1.91
S-69-06	Soil sample; mean grain size 2.4 mm [HX1-Ro_GRAS_MarSCoDe-LIBS-I-06_SCI_N_20210723192057_20210723192118_00069_A.2B]	2.7160 m	9.17	45.04	1.20	0.37	2.69	7.75	18.88	5.28	1.24
S-103-07	Soil sample; mean grain size 2.0 mm [HX1-Ro_GRAS_MarSCoDe-LIBS-I-07_SCI_N_20210827162621_20210827162642_00103_A.2B]	2.5067 m	9.48	40.51	0.74	0.15	7.09	7.75	20.08	3.31	0.94
S-110-06	The first of two consecutive measurements at one soil target; mean grain size 2.1 mm [HX1-Ro_GRAS_MarSCoDe-LIBS-I-06_SCI_N_20210903204535_20210903204556_00110_A.2B]	2.2679 m	6.52	42.94	0.83	0.20	3.51	7.43	13.51	3.42	3.43
S-110-07	The second of two consecutive measurements at one soil target; mean grain size 2.1 mm [HX1-Ro_GRAS_MarSCoDe-LIBS-I-07_SCI_N_20210903205924_20210903205945_00110_A.2B]	2.2731 m	9.70	50.82	1.16	0.33	5.36	9.55	22.69	2.91	2.05
		Soil average (wt%)	8.65	43.13	1.02	0.26	4.67	7.86	18.88	3.39	2.60
		SD (wt%)	1.30	6.54	0.26	0.08	1.73	1.63	3.42	1.07	1.70

TAR-sands											
**Sample ID**	**Descriptions**	**Distance**	**CaO**	**SiO_2**	**Na_2O**	**K_2O**	**MgO**	**Al_2O_3**	**FeO_T**	**TiO_2**	**H_2O**
T-45-06	At the foot of the windward side of the TAR-45; mean grain size 2.4 mm [HX1-Ro_GRAS_MarSCoDe-LIBS-I-06_SCI_N_20210629073102_20210629073123_00045_A.2B]	3.1297 m	11.62	50.21	1.14	0.15	8.91	11.03	14.67	2.07	1.45
T-47-06	Sample of windward side of TAR-45; mean grain size 0.6 mm; dust rich [HX1-Ro_GRAS_MarSCoDe-LIBS-I-06_SCI_N_20210701022936_20210701022957_00047_A.2B]	2.6337 m	10.92	51.82	1.06	0.23	6.18	9.20	28.92	6.38	3.02
T-47-07	Sample of windward side of the TAR-65; mean grain size 0.3 mm; dust rich [HX1-Ro_GRAS_MarSCoDe-LIBS-I-07_SCI_N_20210701024155_20210701024216_00047_A.2B]	2.3373 m	8.98	31.14	0.99	0.22	5.98	6.77	18.79	3.63	0.82
T-65-06	Sample of the ridge of the TAR-65; surface covered by coarse grains, mean size 2.3 mm [HX1-Ro_GRAS_MarSCoDe-LIBS-I-06_SCI_N_20210719164325_20210719164346_00065_A.2B]	2.9832 m	9.30	48.74	0.84	0.27	3.59	8.35	27.95	7.63	0.60
T-65-07	Sample of dark materials on the depression of the TAR-65; covered by coarse grains, mean 1.8 mm; dust rich [HX1-Ro_GRAS_MarSCoDe-LIBS-I-07_SCI_N_20210719165714_20210719165735_00065_A.2B]	3.0263 m	8.89	38.42	0.72	0.16	7.43	7.44	17.82	3.08	6.80
T-92-06	Sample of the ridge of the third TAR; dust covered; grain size unresolvable (< 0.2 mm) [HX1-Ro_GRAS_MarSCoDe-LIBS-I-06_SCI_N_20210816093931_20210816093952_00092_A.2B]	2.5669 m	9.29	53.48	0.91	0.24	4.62	9.90	19.17	3.46	3.17
T-92-07	Sample of the windward side of the third TAR; dust covered; grain size unresolvable (< 0.2 mm) [HX1-Ro_GRAS_MarSCoDe-LIBS-I-07_SCI_N_20210816095320_20210816095341_00092_A.2B]	3.0067 m	8.08	40.92	0.87	0.22	5.36	7.19	16.12	3.40	3.34
		TAR-sand average (wt%)	9.58	44.96	0.93	0.21	6.01	8.55	20.49	4.24	2.74
		SD (wt%)	3.50	17.69	0.24	0.13	2.27	3.13	8.54	2.03	1.68

Unclassified											
**Sample ID**	**Descriptions**	**Distance**	**CaO**	**SiO_2**	**Na_2O**	**K_2O**	**MgO**	**Al_2O_3**	**FeO_T**	**TiO_2**	**H_2O**
R-21-05	Targeted onto the pitted rocks; LIBS laser shot location unresolvable in the micro-image [HX1-Ro_GRAS_MarSCoDe-LIBS-I-05_SCI_N_20210604131244_20210604131304_00021_A.2B]	4.0492 m	19.59	102.56	1.92	0.21	10.85	24.74	19.74	2.29	2.36

R-21-06	Targeted onto the pitted rocks; LIBS laser shot location unresolvable in the micro-image [HX1-Ro_GRAS_MarSCoDe-LIBS-I-06_SCI_N_20210604132442_20210604132502_00021_A.2B]	2.9138 m	8.76	43.40	0.91	0.26	3.55	6.91	16.89	4.18	2.03

Notes. The calibration targets and results are reported as oxides, including H_2O. The unit of the major elements and water is wt%. Brackets in the descriptions indicate the data IDs of the LIBS files. The spectral wavelengths used for deriving the compositions are as follows: Ca II 393.4 nm; Si I 288.16 nm; Na I 589.00 nm; K I 769.9 nm; Mg II 279.6 nm; Al I 394.4 nm; Fe II 259.9 nm; Ti II 334.9 nm; H I 656.3 nm. The root mean square error of calibration (RMSEC) of the quantification model for each element is estimated to be 0.29 wt% H_2O, 0.79 wt% Na_2O, 0.76 wt% MgO, 9.38 wt% Al_2O_3, 22.66 wt% SiO_2, 0.05 wt% K_2O, 2.48 wt% CaO, 0.10 wt% TiO_2, and 1.35 wt% FeO_T. The mean relative error of calibration (REC) of the quantification model for each element is estimated to be 15.62% H_2O, 43.40% Na_2O, 7.33% MgO, 52.57% Al_2O_3, 38.32% SiO_2, 62.08% K_2O, 18.74% CaO, 15.24% TiO_2, and 8.31% FeO_T.

Supplementary Table 2. The IDs for the Tianwen-1 imagery data used in the figures.

Figures	File name in Planetary Data System (Data IDs)
Figure 1C	HX1-Ro_GRAS_NaTeCamA-F-001_SCI_N_20210523023057_20210523023057_00009_A.2CL
	HX1-Ro_GRAS_NaTeCamA-F-002_SCI_N_20210523023232_20210523023232_00009_A.2CL
	HX1-Ro_GRAS_NaTeCamA-F-003_SCI_N_20210523023407_20210523023407_00009_A.2CL
	HX1-Ro_GRAS_NaTeCamA-F-004_SCI_N_20210523023542_20210523023542_00009_A.2CL
	HX1-Ro_GRAS_NaTeCamA-F-005_SCI_N_20210523023717_20210523023717_00009_A.2CL
Figure 2	HX1-Ro_GRAS_NaTeCamA-F-001_SCI_N_20210625233144_20210625233144_00042 A.2CL
	HX1-Ro_GRAS_MarSCoDe-Micro1024-001-06_SCI_N_20210626235327_20210626235327_00043_A.2BL
	HX1-Ro_GRAS_MarSCoDe-Micro1024-001-07_SCI_N_20210627000546_20210627000546_00043_A.2BL
	HX1-Ro_GRAS_NaTeCamA-F-002_SCI_N_20210628005159_20210628005159_00044_A.2CL
	HX1-Ro_GRAS_MarSCoDe-Micro1024-001-06_SCI_N_20210629073213_20210629073213_00045_A.2BL
	HX1-Ro_GRAS_MarSCoDe-Micro1024-001-07_SCI_N_20210629074433_20210629074433_00045_A.2BL
	HX1-Ro_GRAS_NaTeCamB-F-003_SCI_N_20210801213823_20210801213823_00078_A.2CL
	HX1-Ro_GRAS_MarSCoDe-Micro1024-001-06_SCI_N_20210803015246_20210803015246_00079_A.2B
	HX1-Ro_GRAS_MarSCoDe-Micro1024-001-07_SCI_N_20210803020635_20210803020635_00079_A.2B
	HX1-Ro_GRAS_NaTeCamB-F-005_SCI_N_20210823183716_20210823183716_00099_A.2CL
	HX1-Ro_GRAS_MarSCoDe-Micro1024-027-06_SCI_N_20210824142202_20210824142202_00100.2B
	HX1-Ro_GRAS_MarSCoDe-Micro1024-094-07_SCI_N_20210824143552_20210824143552_00100.2B
Figure 3	HX1-Ro_GRAS_NaTeCamA-F-001_SCI_N_20210629001750_20210629001750_00045_A.2C
	HX1-Ro_GRAS_NaTeCamA-F-002_SCI_N_20210629001925_20210629001925_00045_A.2C
	HX1-Ro_GRAS_NaTeCamA-F-003_SCI_N_20210629002100_20210629002100_00045_A.2C
	HX1-Ro_GRAS_NaTeCamA-F-004_SCI_N_20210629002235_20210629002235_00045_A.2C
	HX1-Ro_GRAS_NaTeCamA-F-005_SCI_N_20210629002410_20210629002410_00045_A.2C
	HX1-Ro_GRAS_NaTeCamA-F-006_SCI_N_20210629002545_20210629002545_00045_A.2C
	HX1-Ro_GRAS_NaTeCamB-F-002_SCI_N_20210630015024_20210630015024_00046_A.2CL
	HX1-Ro_GRAS_MarSCoDe-Micro1024-001-07_SCI_N_20210701024306_20210701024306_00047.2B
	HX1-Ro_GRAS_MarSCoDe-Micro1024-001-06_SCI_N_20210701023047_20210701023047_00047.2B
	HX1-Ro_GRAS_NaTeCamA-F-002_SCI_N_20210718125553_20210718125553_00064_A.2C
	HX1-Ro_GRAS_NaTeCamA-F-003_SCI_N_20210718125728_20210718125728_00064_A.2C
	HX1-Ro_GRAS_NaTeCamA-F-004_SCI_N_20210718125903_20210718125903_00064_A.2C
	HX1-Ro_GRAS_NaTeCamA-F-003_SCI_N_20210816102503_20210816102503_00092_A.2C
	HX1-Ro_GRAS_NaTeCamA-F-003_SCI_N_20210718125728_20210718125728_00064_A.2CL
	HX1-Ro_GRAS_MarSCoDe-Micro1024-001-06_SCI_N_20210719164415_20210719164415_00065.2B
	HX1-Ro_GRAS_MarSCoDe-Micro1024-001-07_SCI_N_20210719165806_20210719165806_00065.2B
	HX1-Ro_GRAS_NaTeCamA-F-004_SCI_N_20210816102638_20210816102638_00092_A.2C
	HX1-Ro_GRAS_NaTeCamA-F-005_SCI_N_20210816102813_20210816102813_00092_A.2C
	HX1-Ro_GRAS_NaTeCamA-F-006_SCI_N_20210816102948_20210816102948_00092_A.2C

	HX1-Ro_GRAS_NaTeCamA-F-007_SCI_N_20210816103123_20210816103123_00092_A.2C<br><br>HX1-Ro_GRAS_NaTeCamA-F-001_SCI_N_20210815054820_20210815054820_00091_A.2CL<br>HX1-Ro_GRAS_MarSCoDe-Micro1024-001-06_SCI_N_20210816094022_20210816094022_00092.2B<br>HX1-Ro_GRAS_MarSCoDe-Micro1024-001-07_SCI_N_20210816095411_20210816095411_00092.2B
Supplementary Figure 1	HX1-Ro_GRAS_NaTeCamB-F-004_SCI_N_20210603090654_20210603090654_00020_A.2CL<br>HX1-Ro_GRAS_NaTeCamA-F-006_SCI_N_20210603090947_20210603090947_00020_A.2CL<br>HX1-Ro_GRAS_NaTeCamA-F-012_SCI_N_20210719131643_20210719131643_00065_A.2CL<br>HX1-Ro_GRAS_NaTeCamB-F-001_SCI_N_20210827170411_20210827170411_00103_A.2CL
Supplementary Figure 2	HX1-Ro_GRAS_NaTeCamA-F-002_SCI_N_20210527052907_20210527052907_00013_A.2CL<br>HX1-Ro_GRAS_NaTeCamB-F-006_SCI_N_20210603091004_20210603091004_00020_A.2CL<br>HX1-Ro_GRAS_NaTeCamB-F-003_SCI_N_20210606113359_20210606113359_00023_A.2CL<br>VL2 landing site: jpegPIA00363.jpg<br>HX1-Ro_GRAS_NaTeCamA-F-002_SCI_N_20210628005159_20210628005159_00044_A.2CL<br>HX1-Ro_GRAS_NaTeCamA-F-001_SCI_N_20210615221152_20210615221152_00032_A.2CL
Supplementary Figure 3	Viking 1: PIA03164
Supplementary Figure 4	HX1-Ro_GRAS_NaTeCamA-F-001_SCI_N_20210521002104_20210521002104_00007_A.2CL<br>HX1-Ro_GRAS_NaTeCamA-F-001_SCI_N_20210723153650_20210723153650_00069_A.2CL<br>HX1-Ro_GRAS_NaTeCamA-F-002_SCI_N_20210814052927_20210814052927_00090_A.2CL<br>HX1-Ro_GRAS_NaTeCamA-F-002_SCI_N_20210818105921_20210818105921_00094_A.2CL<br>HX1-Ro_GRAS_NaTeCamA-F-001_SCI_N_20210822124459_20210822124459_00098_A.2CL<br>HX1-Ro_GRAS_NaTeCamA-F-002_SCI_N_20210831185604_20210831185604_00107_A.2CL
Supplementary Figure 5	HX1-Ro_GRAS_NaTeCamA-F-003_SCI_N_20210614164834_20210614164834_00031_A.2CL<br>HX1-Ro_GRAS_MarSCoDe-Micro64-243-05_SCI_N_20210615173823_20210615173823_00032.2B<br><br>HX1-Ro_GRAS_NaTeCamB-F-002_SCI_N_20210623221017_20210623221017_00040_A.2CL<br>HX1-Ro_GRAS_MarSCoDe-Micro1024-001-06_SCI_N_20210625034322_20210625034322_00041.2B<br><br>HX1-Ro_GRAS_NaTeCamB-F-001_SCI_N_20210625233201_20210625233201_00042_A.2CL<br>HX1-Ro_GRAS_MarSCoDe-Micro1024-001-07_SCI_N_20210627000546_20210627000546_00043.2B<br><br>HX1-Ro_GRAS_NaTeCamA-F-004_SCI_N_20210703033927_20210703033927_00049_A.2CL<br>HX1-Ro_GRAS_MarSCoDe-Micro1024-001-06_SCI_N_20210704104856_20210704104856_00050.2B<br>HX1-Ro_GRAS_MarSCoDe-Micro1024-001-07_SCI_N_20210704110116_20210704110116_00050.2B
Supplementary Figure 6	HX1-Ro_GRAS_NaTeCamA-F-003_SCI_N_20210614164834_20210614164834_00031_A.2CL<br>HX1-Ro_GRAS_MarSCoDe-Micro64-231-06_SCI_N_20210615175021_20210615175021_00032.2B<br><br>HX1-Ro_GRAS_NaTeCamB-F-001_SCI_N_20210625233201_20210625233201_00042_A.2CL<br>HX1-Ro_GRAS_MarSCoDe-LIBS-I-06_SCI_N_20210626235216_20210626235237_00043.2B<br><br>HX1-Ro_GRAS_NaTeCamB-F-003_SCI_N_20210801213823_20210801213823_00078_A.2CL<br>HX1-Ro_GRAS_MarSCoDe-Micro1024-001-06_SCI_N_20210803015545_20210803015545_00079_A.2B<br>HX1-Ro_GRAS_MarSCoDe-Micro1024-001-06_SCI_N_20210803015246_20210803015246_00079_A.2B<br>HX1-Ro_GRAS_NaTeCamB-F-003_SCI_N_20210801213823_20210801213823_00078_A.2CL<br>HX1-Ro_GRAS_MarSCoDe-Micro1024-001-07_SCI_N_20210803020934_20210803020934_00079_A.2B<br>HX1-Ro_GRAS_MarSCoDe-Micro1024-001-07_SCI_N_20210803020635_20210803020635_00079_A.2B

	HX1-Ro_GRAS_MarSCoDe-Micro1024-001-07_SCI_N_20210723193537_20210723193537_00069.2B<br>HX1-Ro_GRAS_MarSCoDe-Micro1024-027-06_SCI_N_20210811071102_20210811071102_00087.2B<br>HX1-Ro_GRAS_MarSCoDe-Micro1024-094-07_SCI_N_20210811072451_20210811072451_00087.2B<br>HX1-Ro_GRAS_MarSCoDe-Micro1024-001-06_SCI_N_20210827161323_20210827161323_00103.2B
Supplementary Figure 7	HX1-Ro_GRAS_MarSCoDe-Micro1024-001-06_SCI_N_20210625034322_20210625034322_00041_A.2BL<br>HX1-Ro_GRAS_MarSCoDe-Micro1024-001-07_SCI_N_20210625035541_20210625035541_00041_A.2BL<br>HX1-Ro_GRAS_MarSCoDe-Micro1024-001-07_SCI_N_20210627000546_20210627000546_00043_A.2BL<br>HX1-Ro_GRAS_MarSCoDe-Micro1024-001-06_SCI_N_20210704104856_20210704104856_00050_A.2BL<br>HX1-Ro_GRAS_MarSCoDe-Micro1024-001-07_SCI_N_20210704110116_20210704110116_00050_A.2BL<br>HX1-Ro_GRAS_MarSCoDe-Micro1024-001-07_SCI_N_20210712091102_20210712091102_00058_A.2BL<br>HX1-Ro_GRAS_MarSCoDe-Micro1024-001-08_SCI_N_20210712092452_20210712092452_00058_A.2BL<br>HX1-Ro_GRAS_MarSCoDe-Micro1024-001-06_SCI_N_20210723191849_20210723191849_00069_A.2BL<br>HX1-Ro_GRAS_MarSCoDe-Micro1024-001-07_SCI_N_20210827162413_20210827162413_00103_A.2BL<br>HX1-Ro_GRAS_MarSCoDe-Micro1024-001-06_SCI_N_20210903204327_20210903204327_00110_A.2BL<br>HX1-Ro_GRAS_MarSCoDe-Micro1024-001-06_SCI_N_20210629073213_20210629073213_00045_A.2BL<br>HX1-Ro_GRAS_MarSCoDe-Micro1024-001-06_SCI_N_20210701023047_20210701023047_00047_A.2BL<br>HX1-Ro_GRAS_MarSCoDe-Micro1024-001-06_SCI_N_20210719164118_20210719164118_00065_A.2B<br>HX1-Ro_GRAS_MarSCoDe-Micro1024-001-07_SCI_N_20210719165506_20210719165506_00065_A.2BL<br>HX1-Ro_GRAS_MarSCoDe-Micro1024-001-06_SCI_N_20210816093724_20210816093724_00092_A.2BL

Supplementary Table 3. Chemical composition of selected Martian samples (source data for Figures 3A, 3B).

Martian meteorites [compiled by [12]						
Meteorites	**Types and data sources**	**SiO_2**	**Al_2O_3**	**MgO**	**CaO**	**FeO_T**
Los Angeles	Shergottite group [13]	49.1	11.2	3.53	9.95	21.2
Shergotty	Shergottite group [14]	51.36	7.06	9.28	10	19.41
EETA79001B	Shergottite group [15]	49.03	9.93	7.32	11	17.07
Zagami	Shergottite group [16]	50.5	6.05	11.3	10.5	18.1
ALH77005	Shergottite group [16]	42.4	2.87	28.2	3.16	20.1
Nakhla	Nakhlite group [14]	49.33	1.64	11.82	14.3	21.7
Lafayette	Nakhlite group [16]	46.9	2.47	12.9	13.4	21.6
Chassigny	Chassignite group [16]	37.4	0.72	31.8	0.66	27.3
ALH84001	Orthopyroxenite [16]	52.8	1.29	25	1.82	17.5
In situ measurements of Mars [compiled by [17]]						
In situ selected volcanic and pyroclastic rocks		**SiO_2**	**Al_2O_3**	**MgO**	**CaO**	**FeO_T**
Pathfinder- SFR	Calculated composition of local soil-free-rock at Pathfinder site (weathering rinds included)	57.7	12.3	0.8	6.7	14.2
Pathfinder-Shark-1	Pathfinder site	54.3	10.3	3.7	8.2	15.2
Adirondack	Gusev Plain, Gusev Crater	45.7	10.87	10.83	7.75	18.8
Wishstone	Columbia Hills, Gusev Crater	43.8	15.03	4.5	8.89	11.6
Backstay	Columbia Hills, Gusev Crater	49.5	13.25	8.31	6.04	13
Fastball	Home Plate, Gusev Crater	45.3	7.85	12	5.8	17.8
Bounce Rock	Meridiani Planum	51.6	10.48	6.84	12.09	14.4
In situ selected sedimentary rocks		**SiO_2**	**Al_2O_3**	**MgO**	**CaO**	**FeO_T**
Average Burns Formation	Meridiani Planum	37.1	6.4	7.89	4.98	15.6
Upper Burns (Guadalupe)	Meridiani Planum	36.2	5.85	8.45	4.91	14.8
Lower Burns (Mackenzie)	Meridiani Planum	43	7.27	5.43	4.6	15.6
Peace	Columbia Hills, Gusev Crater	37.3	2.24	21.53	4.9	20.4
Alligator	Columbia Hills, Gusev Crater	41.8	5.49	16.27	4.72	18.3
Posey	Home Plate, Gusev Crater	45.8	9.39	9.56	6.71	15.5
Crawfords	Home Plate, Gusev Crater	46.6	9.98	10.3	6.74	15.4
In situ soil average		**SiO_2**	**Al_2O_3**	**MgO**	**CaO**	**FeO_T**
Basaltic soil [n=15]	Gusev plains, Gusev Crater	46.2	10.1	8.64	6.45	16.3
Dust-rich soil [n=4]	Gusev plains, Gusev Crater	45.9	9.83	8.33	6.27	16.1
Basaltic soil [n=9]	Columbia Hills, Gusev Crater	46.3	10.3	8.67	6.2	15.5
Basaltic soil [n=8]	Meridiani Planum	46.4	9.46	7.29	7.07	18.3
Dust-rich soil [n=4]	Meridiani Planum	45.5	9.22	7.63	6.68	17.9
Hematitic soil [n=13]	Meridiani Planum	39.6	7.86	6.74	5.42	29.5
Pathfinder soil [n=7]	Pathfinder site	42.1	9.5	7.78	6.37	21.6
Utopia soil [n=8]	Viking-Utopia Planitia	47	-	-	6.3	17.5
Chryse soil [n=9]	Viking-Chryse Planitia	47	7.9	6.5	6.2	17.7
Gale* [n=14]	Gale Crater [18]	43.5	9.3	8.27	7.05	18.82
Bagnold [n=12]	Gale Crater [18]	46.38	9.11	9.52	6.91	19.1
Mars Global Soils (MGS)	Gale* and Bagnold not included	45.41	9.71	8.35	6.37	16.73

Supplementary Table 4. Normalization of four target categories against the igneous rock composition (source data for Figure 3C).

Normalization	**CaO**	**SiO_2**	**Na_2O**	**K_2O**	**MgO**	**Al_2O_3**	**FeO_T**	**TiO_2**	**H_2O**
LD/R	0.84	0.53	0.52	0.46	0.62	0.83	0.88	0.67	1.35
CD_avg/R	0.72	0.64	0.65	0.58	0.67	0.66	0.56	0.59	1.97
Soil_avg/R	0.85	0.77	0.66	0.59	1.14	0.95	0.71	0.73	1.97
TAR_avg/R	0.95	0.80	0.60	0.48	1.46	1.04	0.77	0.91	2.07

Notes. “LD” represents lithified duricrusts; “CD_avg”, “Soil_avg” and “TAR_avg” represent average compositions of cemented duricrusts, soils, and TAR sands, respectively.

Supplementary Table 5. Identification and basic information of the in situ LIBS spectra analyzed in this study.

Spectrum ID	Spectrum file name	Sol since landing	Technical status	Target Nature	Target Distance (m)
C1	HX1-Ro_GRAS_MarSCoDe-LIBS-I-01_CAL_N_20210604125723_20210604125744_00021_A.2B	21	Invalid	CAL	1.7102
S1	HX1-Ro_GRAS_MarSCoDe-LIBS-I-05_SCI_N_20210604131244_20210604131304_00021_A.2B		Valid	SCI	4.0492
S2	HX1-Ro_GRAS_MarSCoDe-LIBS-I-06_SCI_N_20210604132442_20210604132502_00021_A.2B		Valid	SCI	2.9138
S3	HX1-Ro_GRAS_MarSCoDe-LIBS-I-05_SCI_N_20210615173712_20210615173733_00032_A.2B	32	Valid	SCI	3.5133
S4	HX1-Ro_GRAS_MarSCoDe-LIBS-I-06_SCI_N_20210615174910_20210615174931_00032_A.2B		Valid	SCI	3.4132
C2	HX1-Ro_GRAS_MarSCoDe-LIBS-I-02_CAL_N_20210625031654_20210625031715_00041_A.2B	41	Valid	CAL	1.6878
S5	HX1-Ro_GRAS_MarSCoDe-LIBS-I-06_SCI_N_20210625034210_20210625034231_00041_A.2B		Valid	SCI	4.2298
S6	HX1-Ro_GRAS_MarSCoDe-LIBS-I-07_SCI_N_20210625035429_20210625035450_00041_A.2B		Valid	SCI	3.3846
C3	HX1-Ro_GRAS_MarSCoDe-LIBS-I-02_CAL_N_20210626232722_20210626232743_00043_A.2B	43	Valid	CAL	1.6878
S7	HX1-Ro_GRAS_MarSCoDe-LIBS-I-06_SCI_N_20210626235216_20210626235237_00043_A.2B		Invalid	SCI	4.0949
S8	HX1-Ro_GRAS_MarSCoDe-LIBS-I-07_SCI_N_20210627000435_20210627000456_00043_A.2B		Valid	SCI	3.1756
S9	HX1-Ro_GRAS_MarSCoDe-LIBS-I-06_SCI_N_20210629073102_20210629073123_00045_A.2B	45	Valid	SCI	3.1297
S10	HX1-Ro_GRAS_MarSCoDe-LIBS-I-07_SCI_N_20210629074321_20210629074342_00045_A.2B		Valid	SCI	2.6978
S11	HX1-Ro_GRAS_MarSCoDe-LIBS-I-06_SCI_N_20210701022936_20210701022957_00047_A.2B	47	Valid	SCI	4.1733
S12	HX1-Ro_GRAS_MarSCoDe-LIBS-I-07_SCI_N_20210701024155_20210701024216_00047_A.2B		Valid	SCI	2.7001
S13	HX1-Ro_GRAS_MarSCoDe-LIBS-I-06_SCI_N_20210704104745_20210704104806_00050_A.2B	50	Valid	SCI	4.1733

S14	HX1-Ro_GRAS_MarSCoDe-LIBS-I-07_SCI_N_20210704110004_20210704110025_00050_A.2B		Valid	SCI	2.7001
S15	HX1-Ro_GRAS_MarSCoDe-LIBS-I-07_SCI_N_20210712091310_20210712091331_00058_A.2B	58	Valid	SCI	2.9216
S16	HX1-Ro_GRAS_MarSCoDe-LIBS-I-08_SCI_N_20210712092659_20210712092720_00058_A.2B		Valid	SCI	2.9454
S17	HX1-Ro_GRAS_MarSCoDe-LIBS-I-06_SCI_N_20210719164325_20210719164346_00065_A.2B	65	Valid	SCI	2.9832
S18	HX1-Ro_GRAS_MarSCoDe-LIBS-I-07_SCI_N_20210719165714_20210719165735_00065_A.2B		Valid	SCI	3.0263
S19	HX1-Ro_GRAS_MarSCoDe-LIBS-I-06_SCI_N_20210723192057_20210723192118_00069_A.2B	69	Valid	SCI	2.7160
S20	HX1-Ro_GRAS_MarSCoDe-LIBS-I-07_SCI_N_20210723193446_20210723193507_00069_A.2B		Valid	SCI	2.8879
C4	HX1-Ro_GRAS_MarSCoDe-LIBS-I-02_CAL_N_20210803012830_20210803012851_00079_A.2B		Valid	CAL	1.6878
S21	HX1-Ro_GRAS_MarSCoDe-LIBS-I-06_SCI_N_20210803015454_20210803015515_00079_A.2B	79	Valid	SCI	1.9108
S22	HX1-Ro_GRAS_MarSCoDe-LIBS-I-07_SCI_N_20210803020843_20210803020904_00079_A.2B		Valid	SCI	1.8332
S23	HX1-Ro_GRAS_MarSCoDe-LIBS-I-06_SCI_N_20210811071011_20210811071032_00087_A.2B	87	Valid	SCI	2.3684
S24	HX1-Ro_GRAS_MarSCoDe-LIBS-I-07_SCI_N_20210811072400_20210811072421_00087_A.2B		Valid	SCI	3.4096
S25	HX1-Ro_GRAS_MarSCoDe-LIBS-I-06_SCI_N_20210816093931_20210816093952_00092_A.2B	92	Valid	SCI	2.5669
S26	HX1-Ro_GRAS_MarSCoDe-LIBS-I-07_SCI_N_20210816095320_20210816095341_00092_A.2B		Valid	SCI	3.0067
C5	HX1-Ro_GRAS_MarSCoDe-LIBS-I-02_CAL_N_20210824135701_20210824135722_00100_A.2B		Valid	CAL	1.6950
S27	HX1-Ro_GRAS_MarSCoDe-LIBS-I-06_SCI_N_20210824142112_20210824142133_00100_A.2B	100	Invalid	SCI	3.2520
S28	HX1-Ro_GRAS_MarSCoDe-LIBS-I-07_SCI_N_20210824143501_20210824143522_00100_A.2B		Valid	SCI	3.1479
S29	HX1-Ro_GRAS_MarSCoDe-LIBS-I-06_SCI_N_20210827161232_20210827161253_00103_A.2B	103	Valid	SCI	2.7767

S30	HX1-Ro_GRAS_MarSCoDe-LIBS-I-07_SCI_N_20210827162621_20210827162642_00103_A.2B		Valid	SCI	2.5067
S31	HX1-Ro_GRAS_MarSCoDe-LIBS-I-06_SCI_N_20210903204535_20210903204556_00110_A.2B	110	Valid	SCI	2.2679
S32	HX1-Ro_GRAS_MarSCoDe-LIBS-I-07_SCI_N_20210903205924_20210903205945_00110_A.2B		Valid	SCI	2.2731

Supplementary Table 6. Major oxide concentrations of the onboard calibration target "Norite".

Oxides	Na_2O	MgO	Al_2O_3	SiO_2	K_2O	CaO	TiO_2	FeO_T
Concentration (wt%)	1.55	9.80	14.66	50.11	0.07	12.96	0.65	16.02
RSD	4.9%	5.3%	0.9%	5.4%	5.5%	5.0%	6.0%	8.1%

Data source: [5].

Supplementary Table 7. Statistics on the total spectral intensities of the baseline-corrected spectra of the CAL and SCI targets before and after normalization, showing the performances of different normalization methods.

	Spectrum type	CAL (4 spectra)			SCI (30 spectra)			Relative bias of means SCI *vs* CAL
		Mean	SD	RSD	Mean	SD	RSD	
Total spectral intensity	Baseline-corrected spectra	1163.9	717.6	61.7%	607.7	394.6	64.9%	-47.8%
	Normalized target distance (m)	3316.6	2042.2	61.6%	4736.1	2420.1	51.1%	42.8%
	Normalized O I 777.2 nm line	658.7	344.1	52.2%	599.7	246.3	41.1%	-9.0%
	Normalized C I 247.9 nm line	909.9	198.7	21.8%	976.4	222.4	22.8%	7.3%

Note. The relative bias of means SCI *vs* CAL is defined as $(Mean_{SCI} - Mean_{CAL})/Mean_{CAL} \times 100$ (%).

Supplementary Table 8. H_2O contents and major oxide concentrations of the samples used as standards for the determination the water contents of the SCI targets.

	Concentrations (wt%)	H_2O	Na_2O	MgO	Al_2O_3	SiO_2	P_2O_5	K_2O	CaO	TiO_2	MnO	FeO_T	S
Rocks	Andesite	1.720	5.090	0.430	15.970	64.120	0.328	3.670	1.230	1.130	0.033	5.960	<0.01
	Rhyodacite	3.920	0.230	0.057	10.940	71.130	0.529	2.290	0.250	0.770	0.003	0.790	3.69
	Trachyandesite	1.000	5.110	2.960	15.500	59.000	0.523	5.180	2.580	0.850	0.104	6.310	<0.01
	Olivine basalt	1.360	3.570	10.050	13.680	43.720	0.661	2.510	8.890	2.400	0.176	12.100	0.02

Notes. The water concentrations were determined by thermogravimetric analysis (TGA); the concentrations of other major oxides were determined by X-ray fluorescence (XRF); the sulfur concentrations were determined by inductively coupled plasma–optical emission spectrometry (ICP-OES).

Supplementary Table 9. Statistics on the intensities of the O I 777.2 nm line of the baseline-corrected spectra of the standards and the SCI targets before and after normalization, showing the effects of normalization with total spectral intensity.

	Spectrum types	Standards (4 spectra)			SCI (30 spectra)			Relative bias means SCI *vs* CAL
		Mean	SD	RSD	Mean	SD	RSD	
Intensity O I 777.2 nm line	Baseline-corrected spectra	2.92	0.627	21.5%	1.17	0.843	71.9%	-59.8%
	Normalized with total spectral intensity	4.94×10^{-3}	0.727×10^{-3}	14.7%	4.13×10^{-3}	1.18×10^{-3}	28.5%	-16.3%

References

1 Xu W, Liu X, Yan Z *et al.* The MarSCoDe Instrument Suite on the Mars Rover of China's Tianwen-1 Mission. *Space Sci. Rev.* 2021; **217:** 64, doi:10.1007/s11214-021-00836-5.

2 Liang X, Chen W, Cao Z *et al.* The Navigation and Terrain Cameras on the Tianwen-1 Mars Rover. *Space Sci. Rev.* 2021; **217:** doi:10.1007/s11214-021-00813-y.

3 Tan X, Liu J, Zhang X *et al.* Design and validation of the scientific data products for China's Tianwen-1 mission. *Space Sci. Rev.* 2021; **217:** 69, doi:10.1007/s11214-021-00843-6.

4 Wan X, Li C, Wang H *et al.* Design, function, and implementation of China's first LIBS instrument (MarSCoDe) on the Zhurong Mars Rover. *At. Spectrosc.* 2021; **42:** 294-298; doi: 10.46770/AS.2021.608.

5 González-Vidal J, Pérez-Pueyo R, and Soneira M. Automatic morphology-based cubic p-spline fitting methodology for smoothing and baseline-removal of Raman spectra. *J. Raman Spectrosc.* 2017; **48**: 878-883, doi: 10.1002/jrs.5130.

6 Cui Z, Jia L, Li L *et al.* A laser-induced breakdown spectroscopy experiment platform for high-degree simulation of MarSCoDe in situ detection on Mars. *Remote Sens.* 2022; **14**: 1954.

7 Peng Y, Zhang L, Cai, Z *et al.* Overview of the Mars climate station for Tianwen-1 mission. *Earth Planet. Physics* 2020; **4**: 371-383, doi:10.26464/epp2020057.

8 Ordonez-Etxeberria I, Hueso R, Sánchez-Lavega A *et al.* Characterization of a local dust storm on Mars with REMS/MSL measurements and MARCI/MRO images. *Icarus* 2020; **338**: 113521, doi: 10.1016/j.icarus.2019.113521.

9 Carr M and Head J. Oceans on Mars: An assessment of the observational evidence and possible fate. *J. Geophys. Res. Planets* 2003; **108**, doi: 10.1029/2002JE001963.

10 Wu X, Liu Y, Zhang C. *et al.* Geological characteristics of China's Tianwen-1 landing site at Utopia Planitia, Mars. *Icarus* 2021; **370**: doi:10.1016/j.icarus.2021.114657.

11 Zhao J, Xiao Z, Huang J *et al.* Geological Characteristics and Targets of High Scientific Interest in the Zhurong Landing Region on Mars. *Geophys. Res. Lett.* 2021; **48:** doi:10.1029/2021gl094903.

12 Martian meterorite compendium, https://curator.jsc.nasa.gov/antmet/mmc/index.cfm

13 Rubin A, Warren P, Greenwood J *et al.* Los Angeles: The most differentiated basaltic martian meteorite. *Geol.* 2000; **28**: 1011-1014.

14 Dreibus G, Palme H, Rammensee W *et al.* Composition of Shergotty parent body: Further evidence of a two component model of planet formation [abstract]. *Lunar Planet. Sci.* XIII 1982; 186-187.

15 Mcsween H and Jarosewich E. Petrogenesis of the Elephant Moraine A79001 meteorite: Multiple magma pulses on the shergottite parent body. *Geochim. Cosmochim.* 1981; **47**: 1501-1513.

16 Lodders K and Fegley B. *The Planetary Scientist's Companion*. New York: Oxford University Press, 1998.

17 Taylor S. and McLennan S. *Planetary Crusts: Their Composition, Origin and Evolution*. Cambridge University Press, 2008.

18 Gellert R. MSL Mars Alpha Particle X-ray Spectrometer 4/5 RDR V1.0 [Dataset] 2013. NASA Planetary Data System. https://doi.org/10.17189/1519440.
